# Development of a deep learning-based surveillance system for forest fire detection and monitoring using UAV

**DOI:** 10.1371/journal.pone.0299058

**Published:** 2024-03-12

**Authors:** Ibrahim SHAMTA, Batıkan Erdem Demir

**Affiliations:** Department of Mechatronics Engineering, Faculty of Technology, Karabük University, Karabük, Türkiye; Mirpur University of Science and Technology, PAKISTAN

## Abstract

This study presents a surveillance system developed for early detection of forest fires. Deep learning is utilized for aerial detection of fires using images obtained from a camera mounted on a designed four-rotor Unmanned Aerial Vehicle (UAV). The object detection performance of YOLOv8 and YOLOv5 was examined for identifying forest fires, and a CNN-RCNN network was constructed to classify images as containing fire or not. Additionally, this classification approach was compared with the YOLOv8 classification. Onboard NVIDIA Jetson Nano, an embedded artificial intelligence computer, is used as hardware for real-time forest fire detection. Also, a ground station interface was developed to receive and display fire-related data. Thus, access to fire images and coordinate information was provided for targeted intervention in case of a fire. The UAV autonomously monitored the designated area and captured images continuously. Embedded deep learning algorithms on the Nano board enable the UAV to detect forest fires within its operational area. The detection methods produced the following results: 96% accuracy for YOLOv8 classification, 89% accuracy for YOLOv8n object detection, 96% accuracy for CNN-RCNN classification, and 89% accuracy for YOLOv5n object detection.

## I. Introduction

Forest fires, increasingly common today, require rigorous scientific exploration of early detection methods. This is crucial to proactively control their spread and mitigate environmental harm [1, 2]. The advent of the Industrial Revolution marked a pivotal moment in Earth’s history, initiating a series of events that significantly altered global fire patterns. This transformation can be attributed to various human-induced factors, including changes in land use and deliberate fire suppression. However, it is essential to recognize that amidst the overall decline in global fire incidence, certain regions worldwide have experienced a notable upswing in fire occurrences over the past five decades. This noticeable shift is substantiated through insightful analysis of historical data and the application of advanced models designed to estimate burned areas [3, 4].

Central Europe has adopted a fire management strategy focusing on fire suppression. However, this approach can harm fuel transportation and connectivity, potentially leading to uncontrolled and more extensive wildfires. Addressing this issue comprehensively requires prioritizing proactive measures over solely relying on firefighting efforts. In alignment with this perspective, the European Union (EU) has developed a 2030 strategy that explicitly recognizes fire prevention as a fundamental element in safeguarding and enhancing the resilience of European forests. Echoing this strategy, the 2022 report from the United Nations Environment Programme underscores the urgent need to allocate resources to prevention, support, and advancing fire protection measures. This marks a shift from reactive strategies to a proactive emphasis on prevention and preparedness [5, 6].

In recent times, integrating Unmanned Aerial Vehicles (UAVs) into various domains has gained significant popularity. UAVs are experiencing a surge in civilian utilization, with applications spanning agricultural spraying, cargo transportation, and aerial filming. Furthermore, their military roles extend to target tracking. Notably, UAVs present numerous advantages, such as cost-effectiveness, reduced risk to human lives, superior safety, and immunity to constraints like human fatigue and operational time limitations [7].

The effectiveness of smoke and fire detection systems that rely on sensors is well-established in closed environments. However, these systems face notable challenges when applied in open areas, such as forests, primarily due to their high cost. Furthermore, these systems are deficient in their capacity to capture essential information visually, a limitation that hampers firefighting teams in their efforts to gauge the present condition of the fire, assess its potential for spreading, and pinpoint the direction of propagation [8, 9].

Interest within the academic area has been directed toward the resolution of early detection, entailing an analysis of the interplay between fire incidents and the corresponding detection mechanisms, the consequences of which have been meticulously scrutinized [10]. Various models were put to the test by researchers to refine the detection of forest fires, where the YOLOvX models notably exhibited a distinctive proficiency, setting them apart from their counterparts within the realm of detection models [11]. The study contributions are as follows:

- Development of a UAV survey system integrating with a Jetson Nano edge and a camera.
- Design and implement a ground station program for the reception and management of information transmitted by the UAV.
- Rigorous testing of YOLO models alongside a comprehensive comparative analysis with the proposed CNN-RCNN model.
- Comparing the results of classification models and object detection models.

Section I introduces the research area, where the existing literature is outlined, and the distinctive contributions made in this study are emphasized. In Section II, a comprehensive review of previous field research is undertaken, focusing on the valuable insights offered by earlier researchers. Section III elaborates on the hardware and methods employed, considering the specific contributions of this study. Section IV is dedicated to discussing the results and the analysis of the algorithms, while Section V summarizes the key findings of this study.

## II. Related works

Wang et al. have presented an enhanced smoke detection model based on YOLOv5. A dataset incorporating natural and synthetic smoke images was compiled, and various loss functions were applied to different YOLOv5 models. The dataset was augmented using the mosaic method, and introducing a dynamic anchor box mechanism addressed inaccuracies in prior information. Furthermore, an attention mechanism was proposed to enhance detection performance. The experimental results demonstrated that the model outperforms traditional deep learning algorithms, achieving a 4.4% higher mean Average Precision (mAP) and a detection speed of 85 FPS, rendering it suitable for engineering applications [12].

Zheng et al. present a novel approach to image denoising with a Hybrid Convolutional Neural Network (HDCNN). HDCNN consists of critical components, including a Dilated Block (DB), RepVGG block (RVB), Feature-refinement Block (FB), and a single convolution. These elements collaborate to enhance denoising. The DB captures context information with dilated convolution, BN, standard convolutions, and ReLU. RVB extracts complementary width features, while FB refines knowledge. A single convolution, combined with residual learning, reconstructs clean images. HDCNN exhibits strong denoising capabilities, as validated through experiments, particularly on public datasets, making it a promising solution for image denoising and blind denoising [13].

Shi et al. introduce a fire monitoring and alarm system based on video surveillance, which offers advantages including rapid response, temperature insensitivity, and accompanying surveillance images. The design incorporates fire and smoke detectors using YOLOv3, trained on a newly created dataset obtained from the internet and labeled using LabelImg. Online Hard Example Mining (OHEM) is employed to address sample imbalance. The fusion of fire and smoke detection results enables the identification of fire alarms. Experimental results using the custom dataset validate the efficacy of the proposed algorithm [14].

Ahmad et al. Presented as a pioneering approach in Content-Based Image Retrieval (CBIR), CBIR-SMANN stands out for its systematic framework. The technique initiates with meticulous preprocessing steps, encompassing image resizing and Gaussian filtering, paving the way for extracting salient points utilizing the Hessian detector. Subsequently, the method computes statistical features such as skewness, mean, kurtosis, and standard deviation. These extracted features are then channeled into an Artificial Neural Network (ANN) for interpolation, facilitating the storage of results in a structured database to enable efficient retrieval. During testing, query images undergo similar preprocessing, enabling feature extraction for comparison within the ANN, which, in turn, retrieves analogous photos from the database. Noteworthy is CBIR-SMANN’s implementation in Python, where it notably achieves an impressive high recall rate of 78% and remarkably swift retrieval times of 980 milliseconds. This surpasses prior methodologies, underscoring its potential for advancing image retrieval techniques. Particularly intriguing is its capacity for future enhancements through the integration of convolutional neural networks [15].

Wang et al. conducted a comprehensive interdisciplinary experiment in Machine Vision, combining digital image processing, machine learning, and deep learning for forest wildfire detection. Challenges in accessibility for students and the quest for higher detection accuracy persist despite advancements in wildfire detection research. The study focused on two core modules: wildfire image classification and wildfire region detection. They proposed a novel algorithm, Reduce-VGGNet, for image classification, achieving 91.20% accuracy, and an optimized CNN model for region detection, reaching 97.35% accuracy. The framework meets the demand for comprehensive Machine Vision experiments but also aids in cultivating talent in machine vision for artificial intelligence. However, adapting the framework for satellite images, known for their data complexity and noise, remains challenging. Future work involves exploring alternative CNN-based methods, refining pre-processing techniques, and incorporating multi-sensor data for enhanced wildfire detection in noisy satellite images [16].

Zhang et al. present a solution to the challenge of timely forest wildfire detection using UAVs equipped with cameras. They address the limitations of constrained UAV image sampling and visual angle restrictions, introducing the FT-ResNet50 model. This model utilizes transfer learning to fine-tune the ResNet network to identify forest fires in UAV-captured imagery. The experimental findings demonstrate the FT-ResNet50 model’s notable performance, achieving a recognition accuracy of 79.48%. This outperforms the ResNet50 and VGG16 models by 3.87% and 6.22%, respectively. The research underscores the significance of image processing in forest fire detection and highlights the FT-ResNet50 model’s superior adaptability, especially when trained with limited labeled UAV images [17].

He et al. devised two simplified YOLOv5-based wildfire detection models specifically for embedded terminals with limited computational capacity, focusing on wildfire detection near power transmission lines to ensure power security. These streamlined models reduce parameters while upholding high accuracy and recall rates for real-time monitoring. Additionally, they introduced a customized wildfire detection model for mobile and embedded platforms, mainly targeting transmission lines. In rigorous experiments with a dataset of 1993 images, the first improved YOLO wildfire detection algorithm achieved an average accuracy of 71.5% and a recall rate of 66.2%, outperforming other algorithms by an average of 4.2 percentage points in Average Precision (AP). The second enhanced YOLO model, with a compact 0.9 Mb size, sustained an average accuracy of 64.2% and a recall rate of 63.0%, enabling real-time monitoring on embedded platforms. These models offer crucial insights into wildfire detection near transmission lines while adeptly addressing the limitations of embedded systems, significantly contributing to the safety and security of power infrastructure [18].

Zhan et al. confront the critical challenge of accurately detecting forest fire smoke, an essential component in forest fire prevention and management. They highlight the struggle of current detection methods in capturing the unique traits of smoke, such as its high transparency and indistinct edges, resulting in reduced accuracy. Introducing ARGNet, the paper proposes a novel approach that combines various elements to improve smoke detection significantly. Experimental results on the UAV-IoT platform showcase ARGNet’s exceptional performance, offering a low parametric count and delivering impressive metrics: a mean Average Precision (mAP) of 79.03%, mAP50 of 90.26%, mAP75 of 82.35%, operating at 122.5 FPS with 55.78 GFLOPs. Compared to mainstream methods, ARGNet stands out by enabling real-time detection with high accuracy, proving to be a valuable asset in forest fire prevention and management efforts. This research significantly contributes to securing forestry resources and ensuring ecological safety by enhancing smoke detection in forest fire scenarios [19].

Following an extensive review of prior studies in this field, this research introduces an innovative solution—a sophisticated, intelligent monitoring system designed for early detection of forest fires. This system operates autonomously using a locally manufactured UAV equipped with an NVIDIA Jetson Nano development board for onboard data processing. A specialized CNN-RCNN algorithm significantly enhances the precision and efficiency of image analysis and classification, substantially improving the effectiveness of fire monitoring. Emphasizing an integrated setup, the study combines a ground station interface with an on-board UAV surveillance system. This integrated system transmits critical information to dedicated forest monitoring and civil defense center applications. This collaboration enables real-time, high-precision tracking of fire propagation and furnishes accurate coordinates of the fire’s location, facilitating well-informed decision-making. Leveraging Deep Neural Network algorithms, this system presents a proactive solution to the urgent challenge of forest fire detection and monitoring, which is crucial for environmental conservation and public safety provides a summary of Table 1 [20] the literature review studies.

**Table 1:** Summary of literature review.

Ref.	Model	Application	Results
[11]	YOLOv3, YOLOv5, and YOLOv7	Forest Fire Detection	YOLOv5x with 96.8% accuracy
[12]	YOLOv5x	Smoke Detection	Traditional deep learning algorithms with a 4.4% higher mAP and a detection speed of 85 FPS
[14]	YOLOv3	Fire Monitoring System	The detection speed is greater than 30 FPS
[15]	CBIR-SMANN	Image processing	High recall rate of 78% and a fast retrieval time of 980 ms
[16]	Optimized CNN	Forest Fire Detection	Accuracy is 97.35%
[17]	FT-ResNet50	Forest Wildfire Detection	FT-ResNet50’s superiority with a recognition accuracy of 79.48%, surpassing ResNet50 and VGG16 by 3.87% and 6.22%
[18]	YOLOv5	Forest Fire Detection	Accuracy of 71.5% and a recall rate of 66.2%, surpassing other algorithms by 4.2 percentage points on average precision (AP)
[19]	ARGNet	Forest Fire Smoke Detection	mAP of 79.03%, mAP50 of 90.26%, mAP75 of 82.35%, 122.5 FPS, and 55.78 GFLOPs

## III. Materials and method

This study uses a four-rotor UAV with four propellers to obtain images from the forest for fire detection. Equipped with a camera and an artificial intelligence computer, the UAV performs autonomous flights in a designated area within the forest. During the flight, they embedded deep learning algorithms in the NVIDIA Jetson Nano AI (Artificial Intelligence) computer on the UAV to detect fires from the images captured by the camera. After detecting fire in the images, the corresponding image and coordinate information for that point are transferred to the ground station. Using a developed interface for the ground station, the image and coordinate information related to the fire are presented to the user.

**Fig 1:**
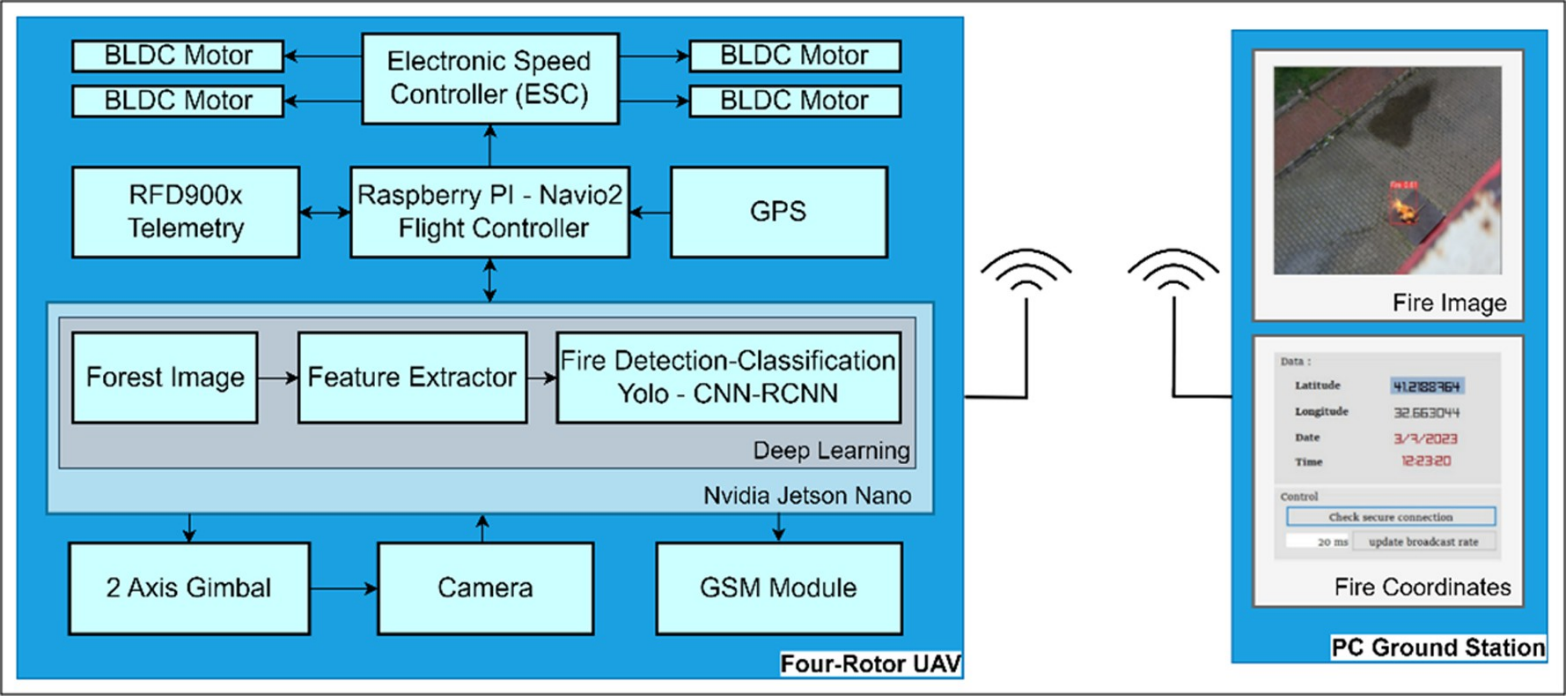
Block diagram of the system.

The proposed system consists of a four-rotor UAV, NVIDIA Jetson Nano AI computer, camera, control and navigation unit, Raspberry Pi small single-board computer and Navio2 flight control board, and GPS/GSM module. The block diagram of the proposed system is provided in Fig 1.

### A. Four-rotor UAV

The four-rotor UAV’s design considered the vehicle’s maximum flight weight and flight time. A fuselage design was created with a calculated maximum flight weight of 2.45 kg and a motor-to-motor diameter of 450 mm, accommodating propellers up to 12 inches in size. The electronic equipment of the four-rotor UAV includes a flight control card, control receiver, telemetry, GPS antenna, power distribution board, power module, ESC, motor, gimbal, and battery. The chosen flight control board for this UAV is the Navio2, which features a high-resolution barometer and two IMU sensors working in conjunction with the Raspberry Pi. The control receiver used is the X8R produced by FRSKY. To receive signals from GPS, GLONASS, Beidou, and Galileo satellites, the Tallysman brand Tw4721 model GNSS antenna is employed. The power distribution board, provided by Matek, ensures efficient power distribution. BLDC motors in the form of SunnySky X3108s motors are utilized, with motor speed adjustment using EMAX BLHeli 25A ESCs. The chosen battery is the Leopard Power 3S 11.1V 6200mAh Li-po battery, enabling a maximum flight time of 25 minutes. The ground station uses the Mission Planner program to fine-tune the flight parameter settings of the four-rotor UAV and to display real-time flight information. Table 2. presents the flight test specifications,

**Table 2:** UAV test specifications.

Flight test specifications	Value
Time	10–15 minutes
Hight	20–30 m
Speed	350 m/s
The angle of the camera	45 degrees
Frame per second of camera	90 FPS

### B. NVIDIA Jetson Nano embedded AI computer

**Fig 2:**
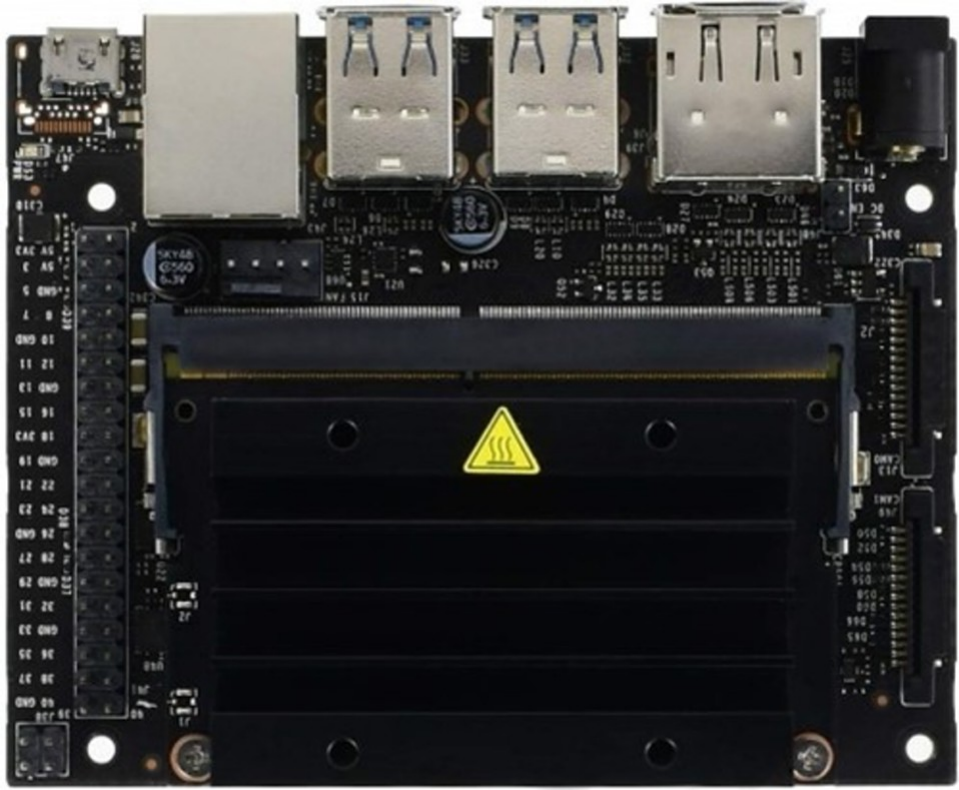
Nvidia Jetson Nano embedded AI computer.

The NVIDIA Jetson Nano developer kit, shown in Fig 2, is a compact yet powerful computer that enables parallel execution of multiple neural networks for applications such as image classification, object detection, segmentation, and speech processing.

All these tasks are performed on an easy-to-use platform with a power consumption of less than 5 watts. It features a Maxwell GPU with a 128-core GPU, end-to-end lossless compression, and texture caching, supporting OpenGL 4.6, OpenGL ES 3.2, Vulkan 1.1, and CUDA. The maximum OpenGL ES shader performance is 512 GFLOPS (FP16) with a maximum operating frequency of 921MHz. The processor is an ARM Cortex-A57 MPCore with NEON Technology (Quad-Core), with 48KB L1 instruction cache per core and 32KB L1 data cache per core. It also has a 2MB L2 Unified Cache, and its maximum operating frequency is 1.43 GHz [21].

### C. Deep learning based image processing

**Fig 3:**
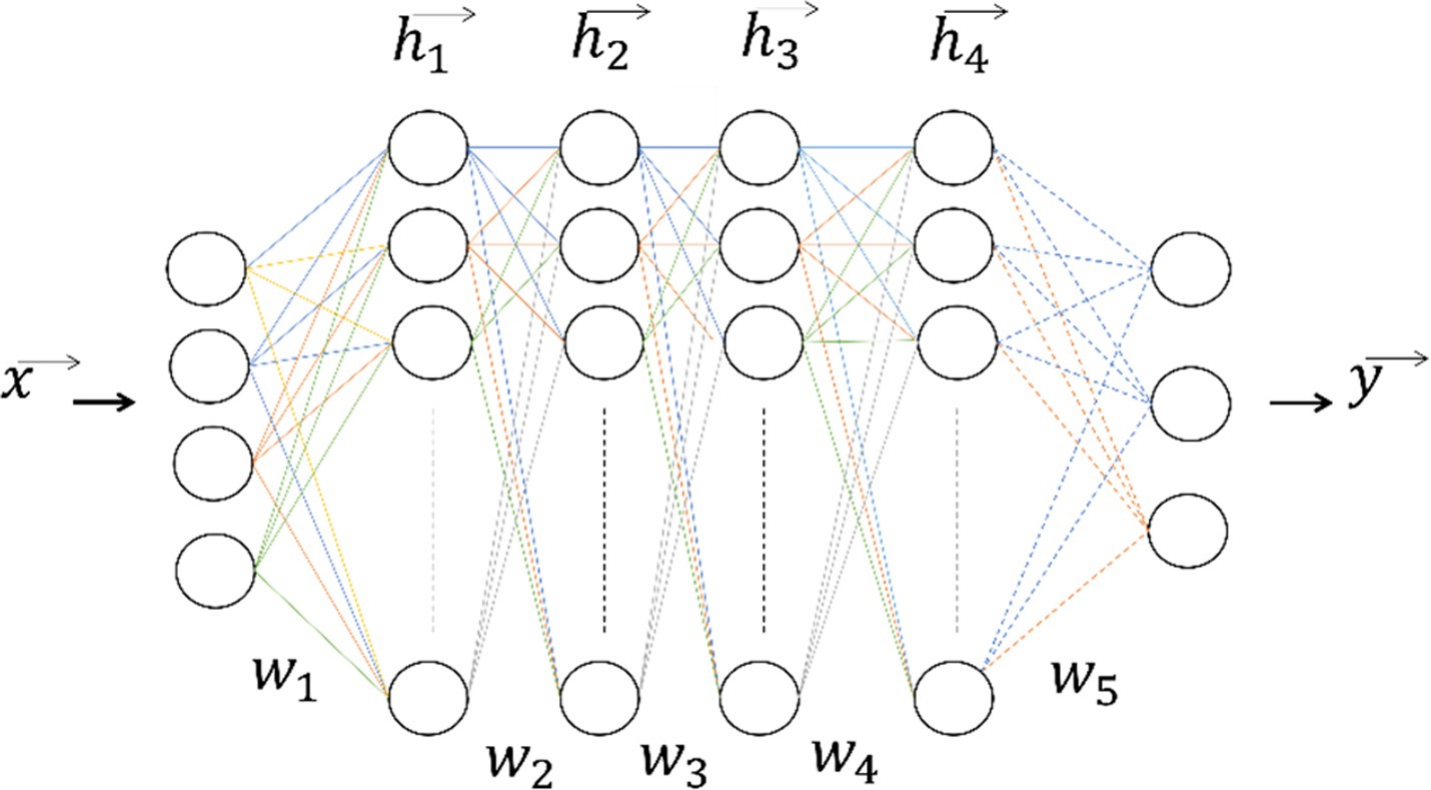
The structure of deep learning.

Deep learning focuses on acquiring suitable data representations to achieve desired outcomes. The term “deep” in deep learning signifies learning hierarchical concepts directly from raw data [22]. It is a class of machine learning that relies on artificial neural networks. The structure of deep learning consists of an input layer, hidden layers, and an output layer, with each layer sequentially serving as the input for the subsequent layer [23]. The structure of deep learning is given in Fig 3. Unlike traditional machine learning, where engineers can manually adjust predictions, the accuracy of forecasts in deep understanding is determined by the underlying algorithms [24].

Several algorithms have been developed specifically for object detection in convolutional neural networks, yielding highly successful outcomes. These algorithms encompass a range of models, such as AlexNet, ZFNet, VGGNet, GoogLeNet, Microsoft ResNet, R-CNN, Fast R-CNN, SSD, and YOLO [25].

### D. Convolutional Neural Networks

**Fig 4:**
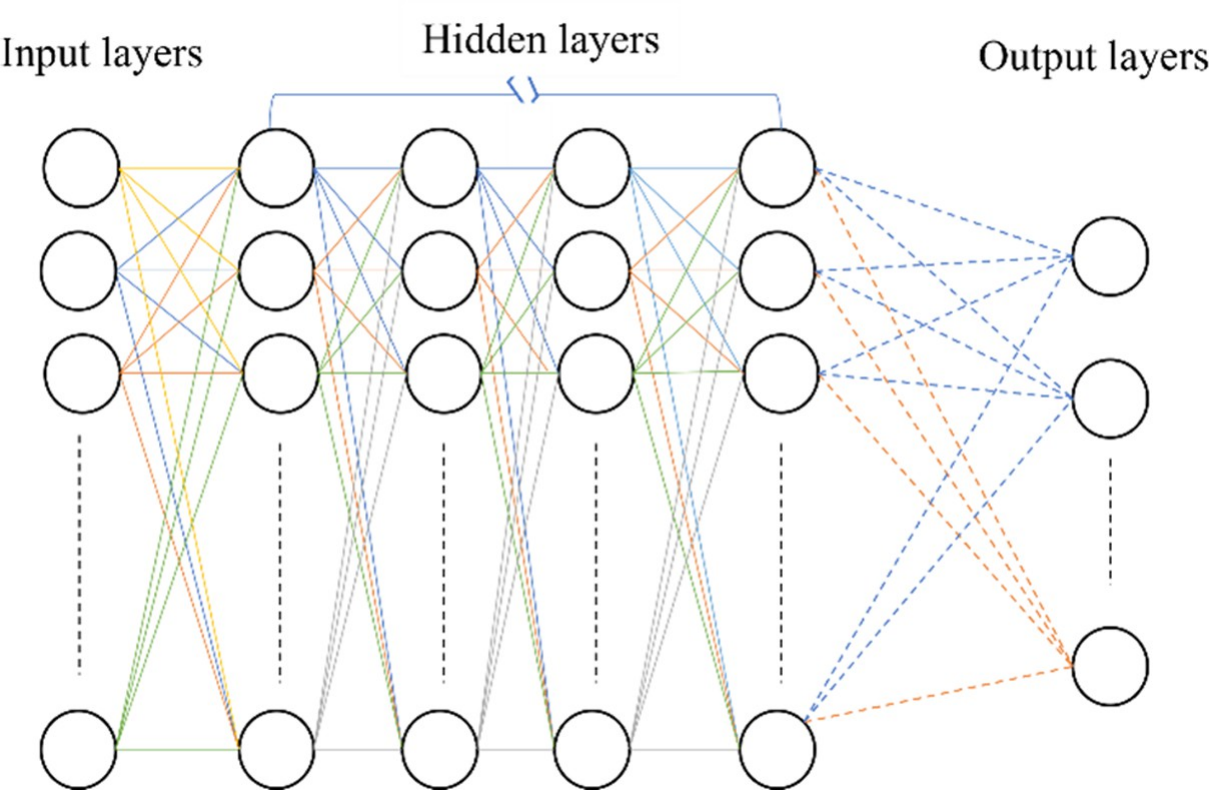
Architecture of CNN.

A Convolutional Neural Network (CNN) is commonly used for image processing and computer vision tasks. It is designed to learn and extract relevant features from images automatically. CNN consists of convolutional layers that apply filters to input images to detect patterns, followed by pooling layers to reduce spatial dimensions and increase computational efficiency. These layers are typically stacked with fully connected layers for classification or regression [26, 27]. Fig 4 shows the architecture of the CNN.

### E. Recurrent Neural Networks

**Fig 5:**
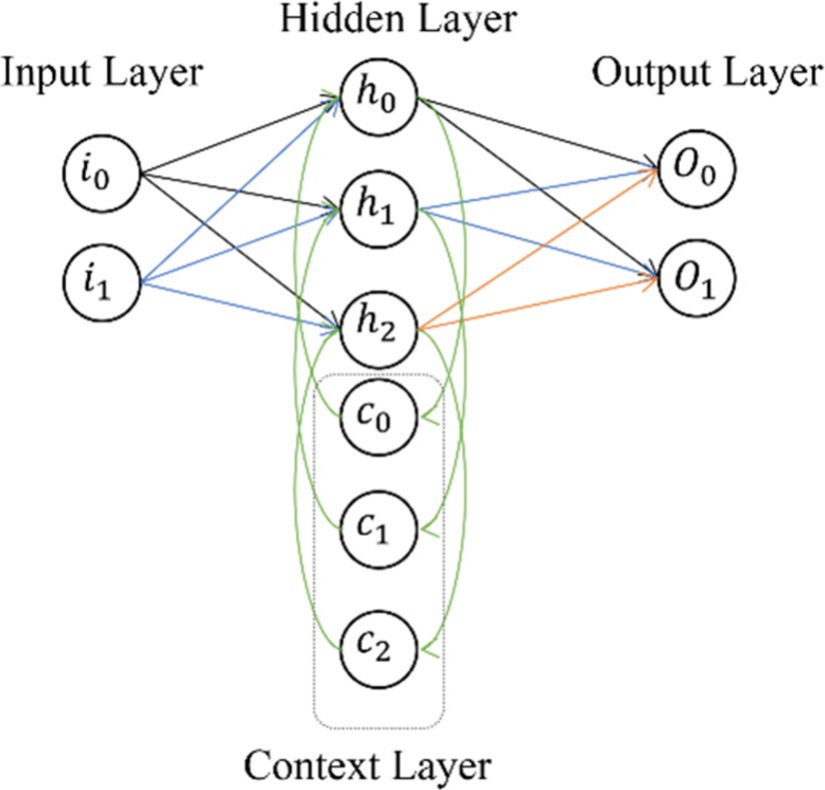
Architecture of RNN.

A Recurrent Neural Network (RNN) is one of the fundamental network architectures on which other deep learning architectures are built. The key difference between a typical multi-layered network and a recurrent network is that a recurrent network can have feedback connections to previous layers (or the same layer) instead of purely feedforward connections, as shown in Fig 5.

This feedback allows RNNs to maintain memory of past inputs and model temporal problems over time. RNNs have a rich set of architectures (such as LSTM—Long Short-Term Memory). The main differentiator is the presence of feedback within the network, which can originate from the hidden layer, output layer, or a combination of both. RNNs can be trained by unfolding them over time and using standard backpropagation or a variant known as Backpropagation Through Time (BPTT) [28].

### F. CNN-RCNN model

The CNN-RCNN model combines the strengths of both CNN and RCNN. It leverages the ability of CNNs to extract spatial features from images and the ability of RCNNs to capture temporal dependencies in sequences [29, 30]. In this study, the model architecture includes convolutional layers for feature extraction from images, followed by recurrent layers (LSTM and GRU–Gated Recurrent Units) to process the extracted features sequentially. The model is designed to handle image classification tasks focusing on fire detection.

**Fig 6:**
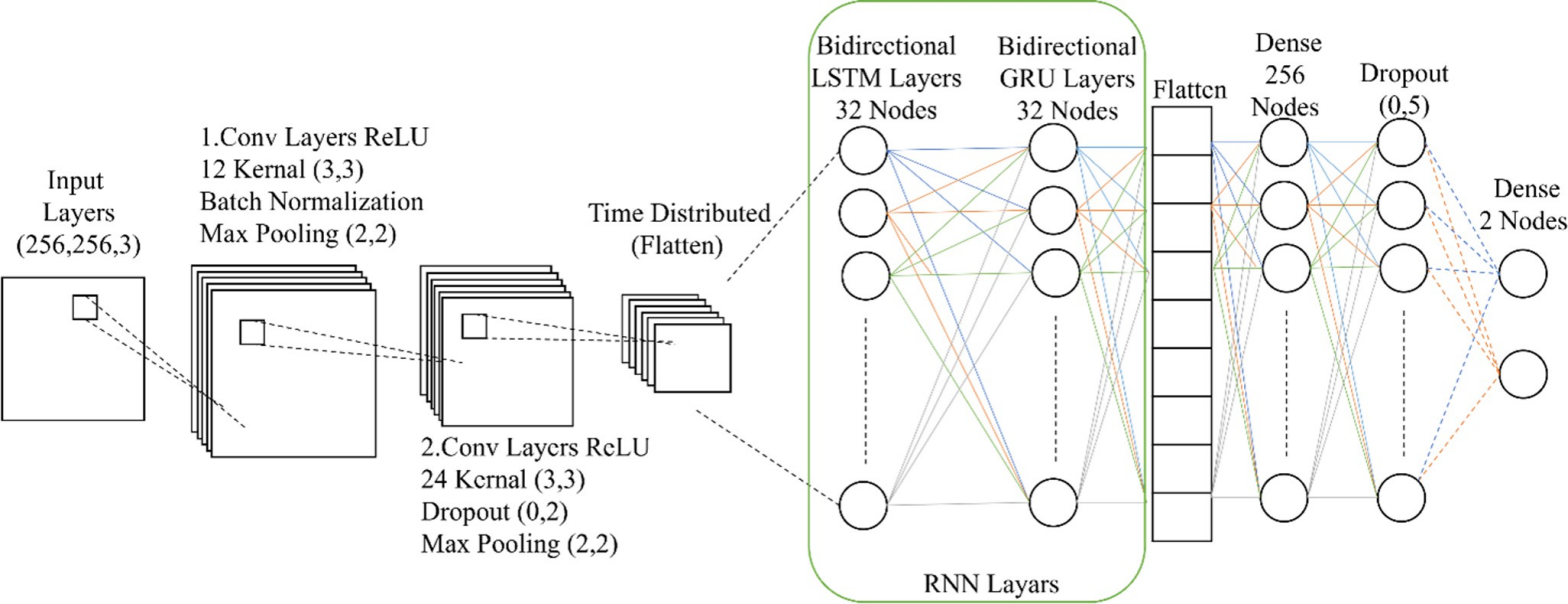
CNN-RCNN model.

The CNN-RCNN model in the code has a unique architecture compared to traditional CNNs or RCNNs. It starts with a CNN component consisting of convolutional layers, batch normalization, and max pooling to extract spatial features from images. Next, it incorporates recurrent layers (LSTM and GRU) within a time-distributed layer, allowing the model to learn sequential patterns from the extracted features. Finally, the model includes dense layers with dropout regularization for classification. This combination of CNN and RCNN components allows the model to effectively learn spatial and temporal features, making it suitable for tasks requiring capturing information from both dimensions. Fig 6 shows the geometric shape of the model. It is worth noting that the success and effectiveness of the CNN-RCNN model depend on various factors, including the quality and diversity of the training data, the specific problem being addressed, and the hyperparameters and architecture choices made in the code.

The model relies on a bidirectional sequential neural network and utilizes convolutional layers, LSTM, and GRU layers for image classification. The convolutional and max pooling layers extract features from the image, while the LSTM and GRU layers handle the temporal information in the moving images. The activation function “relu” is used in the hidden layers, and the “softmax” function is used in the final layer for multi-class classification.

The distinguishing feature of Model CNN-RCNN is its incorporation of LSTM and GRU layers, facilitating the adept handling of temporal information within dynamic visual sequences. This attribute renders it highly suitable for classifying dynamic visual content, notably videos and motion pictures, where the nuanced analysis of temporal dynamics is of paramount importance.

The study meticulously adjusts critical hyperparameters to enhance the forest fire detection model’s efficacy. Data augmentation techniques, such as shear range, zoom range, brightness range, rotation range, and horizontal and vertical flipping, are fine-tuned, facilitating a more diverse and comprehensive training dataset. Within the CNN-RCNN architecture, two convolutional layers with 12 and 24 filters, max-pooling layers of size (2, 2), and recurrent layers integrating Bidirectional LSTM and Bidirectional GRU units, each with 32 units, are implemented. This configuration enriches the model’s capacity to capture sequential information efficiently. Moreover, the model includes a densely connected layer with 256 units and a dropout rate 0.5 for preventing overfitting. The final output layer comprises two units, applying softmax activation. The model is compiled using the Adam optimizer with Categorical Crossentropy as the loss function, fostering effective training. The research underscores the flexibility in training epoch adjustments and incorporates an early stopping mechanism based on validation loss for optimal training outcomes. The dataset is segregated into 80% training and 20% testing subsets, loaded and preprocessed using ImageDataGenerator and label encoding techniques. Post-training evaluation encompasses crucial metrics such as confusion matrices, classification reports, and accuracy assessment, pivotal in understanding the model’s proficiency in forest fire image classification tasks. This systematic approach, carefully tuning hyperparameters and methodically structuring the model, provides a compact yet comprehensive strategy for efficient forest fire detection.

Selecting optimal hyperparameters is a complex challenge in deep learning, given the absence of definitive guidelines for their selection. It demands significant experience and depends on various factors such as the model type, data characteristics, size, and processing techniques. To address this, sure researchers have proposed algorithms that automatically select suitable hyperparameters based on previous model performances [31]. This study employed a combined approach for tuning hyperparameters, leveraging insights from prior models and relying on the trial-and-error method. The details of the hyperparameters utilized in the tuning process are presented in Table 3. This hybrid strategy, integrating insights from established models and iterative refinement through trial and error, was instrumental in optimizing the model’s performance in this research context.

**Table 3:** Hyperparameters of the proposed models.

Hyperparameters	Value
Batch size	32
Learning rate	0.001
Kernal size	(2,2)
Epoach	500

### G. YOLO algorithm

**Fig 7:**
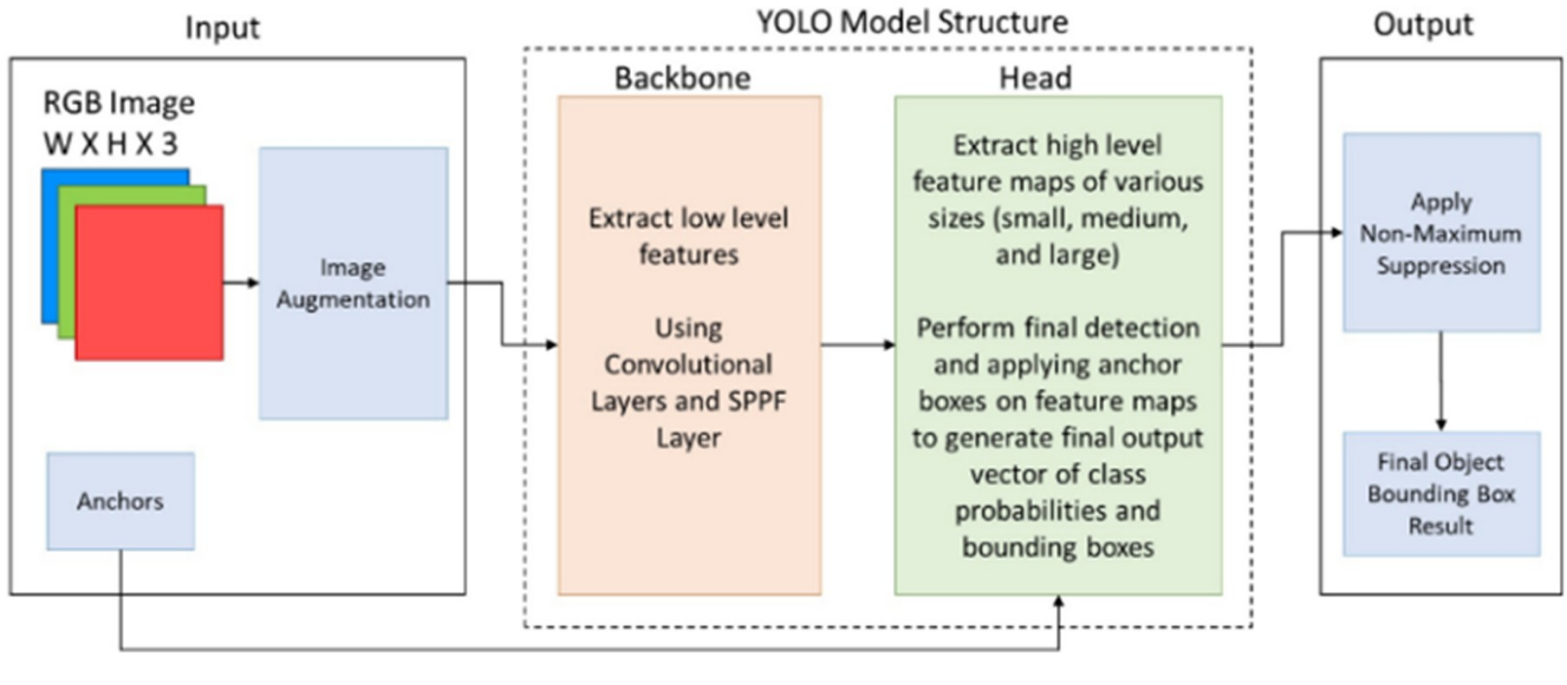
General structure of YOLO.

The YOLO (You Only Look Once) algorithm is among the most powerful and innovative algorithms in object detection in images and videos. YOLO algorithms excel in real-time object detection with their ability to perform high-speed detection, making them one of the fastest algorithms used in computer vision. YOLO demonstrates exemplary performance in detecting objects of variable sizes and dense environments, although it may encounter some difficulties detecting tiny objects. Additionally, YOLO utilizes an output format called “Bounding Box” to represent and determine the locations of detected objects. The YOLO algorithm was initially developed by researcher Joseph Redmon and his team at Washington University in 2016, and since then, it has been continuously improved. The General structure of YOLO architecture for object detection is shown below in Fig 7 [32].

**Fig 8:**
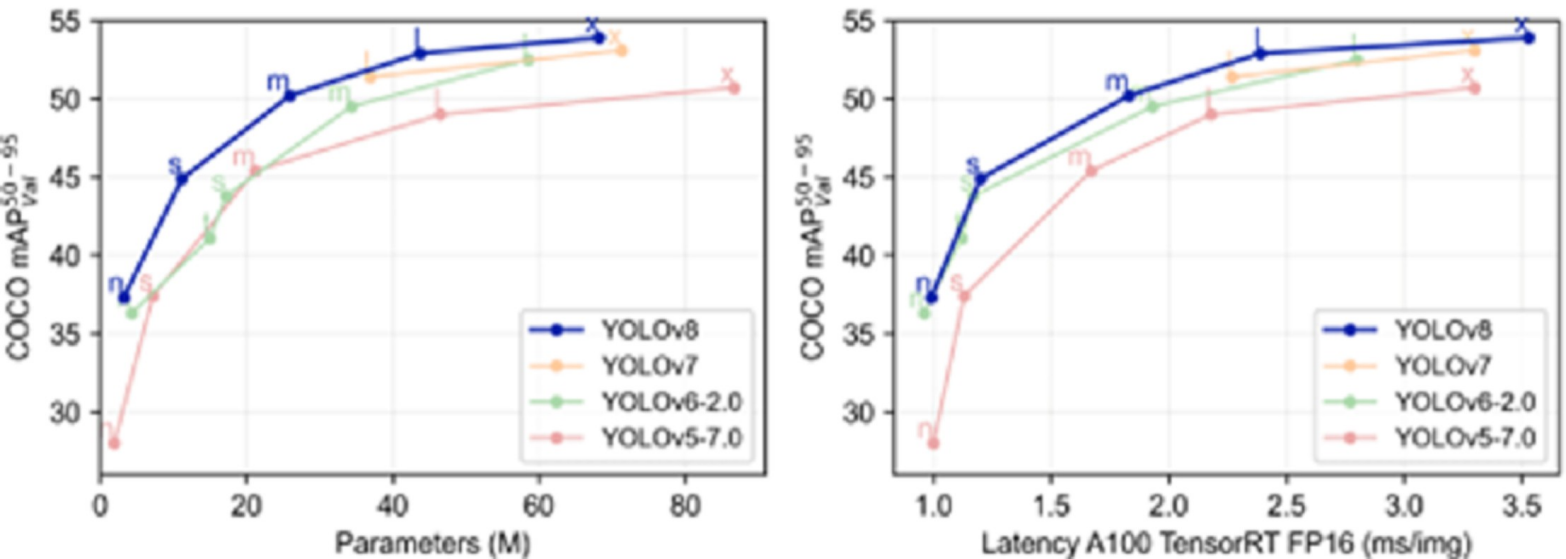
Comparison of YOLOv8 and old versions.

The sequential development of YOLO is as follows: YOLOv1, this version is considered the first iteration for object detection using artificial neural networks, introduced through a research paper published in 2016. The algorithm employs an approach that divides the image into grid cells and then makes predictions for each cell in a single pass. However, this approach faces challenges in detecting small objects and objects with a high degree of occlusion (hidden or partially obscured objects) [33]. YOLOv2, the second version, was released in 2017 through a research paper. In this version, the performance of detecting small objects and handling occlusions was significantly improved. The Darknet-19 network was incorporated, and combining ImageNet and COCO databases enhanced the detection and classification accuracy. This version utilizes Self-Similarity Pyramid Pooling to handle objects of different scales effectively [34–36]. YOLOv3, the third version, was introduced in 2018 through a research paper. In this version, new layers were added for detailed detection, significantly improving the speed and accuracy of detection. Multiple-scale predictions were incorporated within the network to enhance detection at a wider range of scales. The 53-layer Darknet is the underlying model for object detection and classification in this version [37]. YOLOv4, the fourth version, was released in 2020. This version combines techniques from previous iterations, such as multiple-scale detection and additional detail layers. Darknet-53 and enhanced CSPDarknet53 layers are utilized for object detection and classification. YOLOv4 incorporates techniques such as Mish Activation, CIOU Loss, SAM, and PANet to improve performance, resulting in significant advancements in detection speed and accuracy compared to previous versions. YOLOv5, the fifth version of YOLO, was released in 2020 by Glenn Jocher and others. It is not an official continuation of the earlier versions but rather an independent implementation based on PyTorch. A new network architecture called YOLOv5s was utilized, which was smaller and faster than YOLOv4. Additionally, new features were introduced, including automatic model scaling, label smoothing, class probability calibration, and model ensembling. YOLOv5 achieved a mAP of 88.9% on the COCO dataset and operated at 140 FPS on GPU [40]. YOLOv8’s advanced architecture enhances detection accuracy by combining high-level features and contextual information with the C2f module. With its reference-free model structure, it independently handles object presence, classification, and regression tasks, leading to improved overall accuracy. It improves object detection performance, especially for small objects, by employing CIoU and DFL loss functions. Additionally, YOLOv8 introduces a semantic segmentation model with the C2f module and CSPDarknet53 feature extractor. It operates at a high speed of 280 FPS on NVIDIA A100 and TensorRT, showcasing its fast and efficient performance. YOLOv8 is an impressive algorithm with enhanced architecture, reference-free model structure, improved loss functions, semantic segmentation capability, and high speed and efficiency features. According to Ultralytics official website, the developers of YOLOv5 are also the developers of YOLOv8. YOLOv8 is stated as the latest version of this algorithm [38]. We notice in Fig 8, the superiority of YOLOv8 over its peers from previous versions.

### H. Proposed detection and classification model

**Fig 9:**
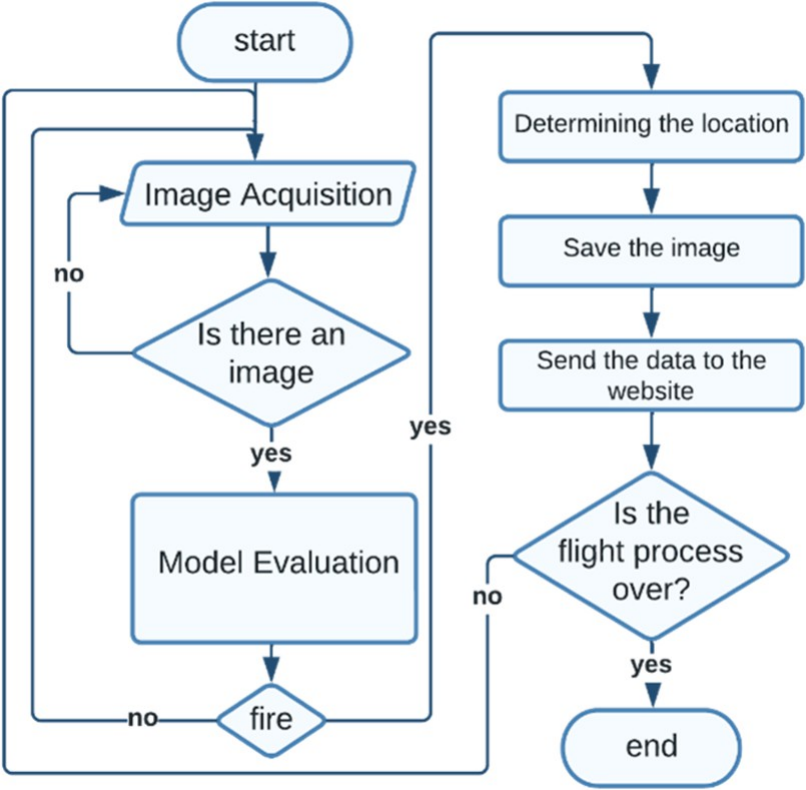
Flowchart diagram of the proposed model.

A block diagram of the proposed model is given in Fig 9, where the process of wildfire detection begins after defining the operational area for the UAV to perform an automatic survey of the area. The NVIDIA Jetson Nano development kit reads the data received from the camera, and the verification process is carried out using an algorithm. Following detection, the data concerning the fire’s exact location and a visual depiction of the fire are expeditiously conveyed to a specialized ground station interface meticulously tailored to present forest fire monitoring results.

**Fig 10:**
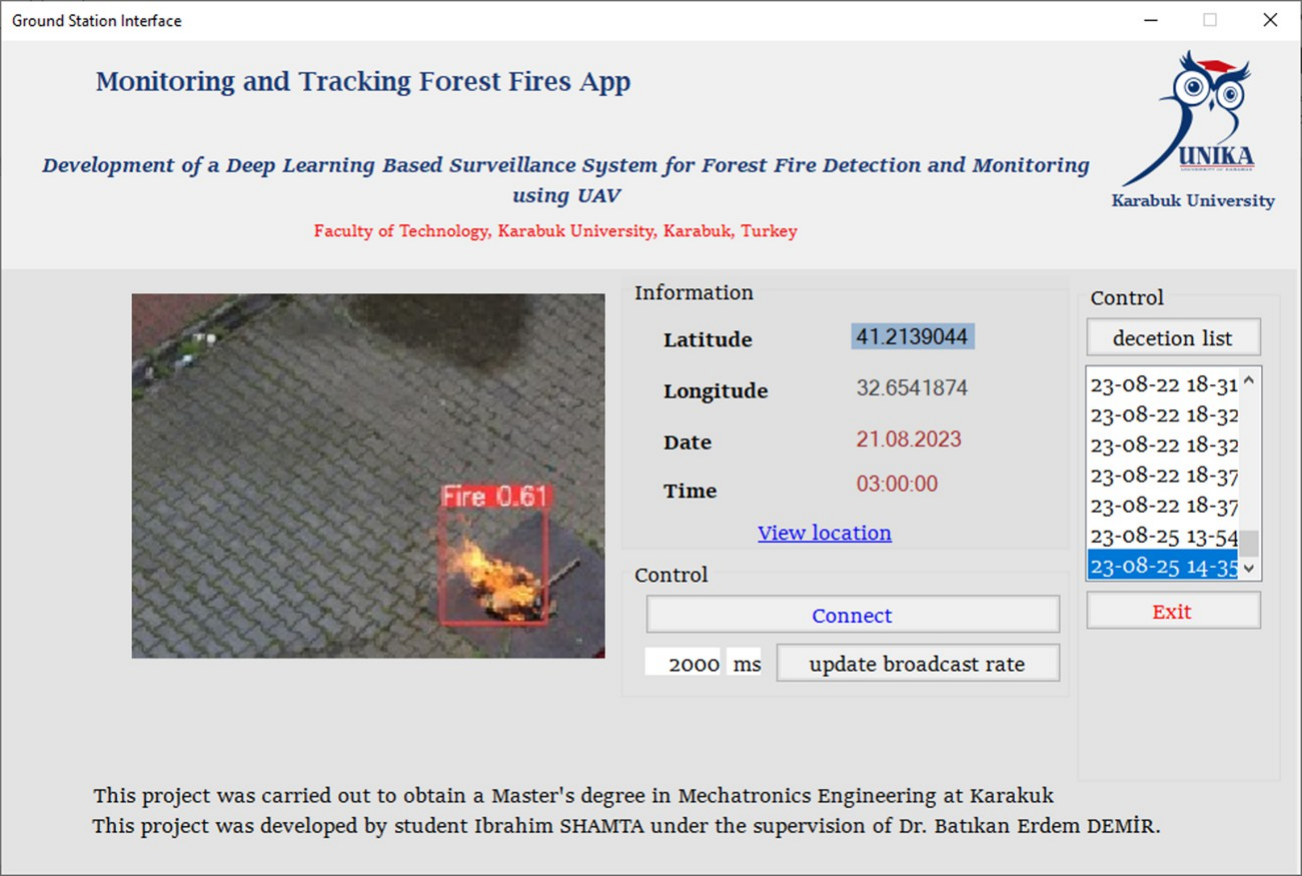
Ground station interface.

The study introduces a specialized ground station interface program to monitor aircraft flying over forested regions. Fig 10 illustrates the ground station interface, which, in the event of forest fire detection, presents the latest aerial images captured by the aircraft and pinpoints the fire’s location through coordinates. It further supplies supplemental data, including the date and time of fire detection. Developed using the C# programming language, this software is intricately linked with a tailored website dedicated to the project. The aircraft’s detection system transmits data to this website, allowing the ground station software to access the most current information.

**Fig 11:**
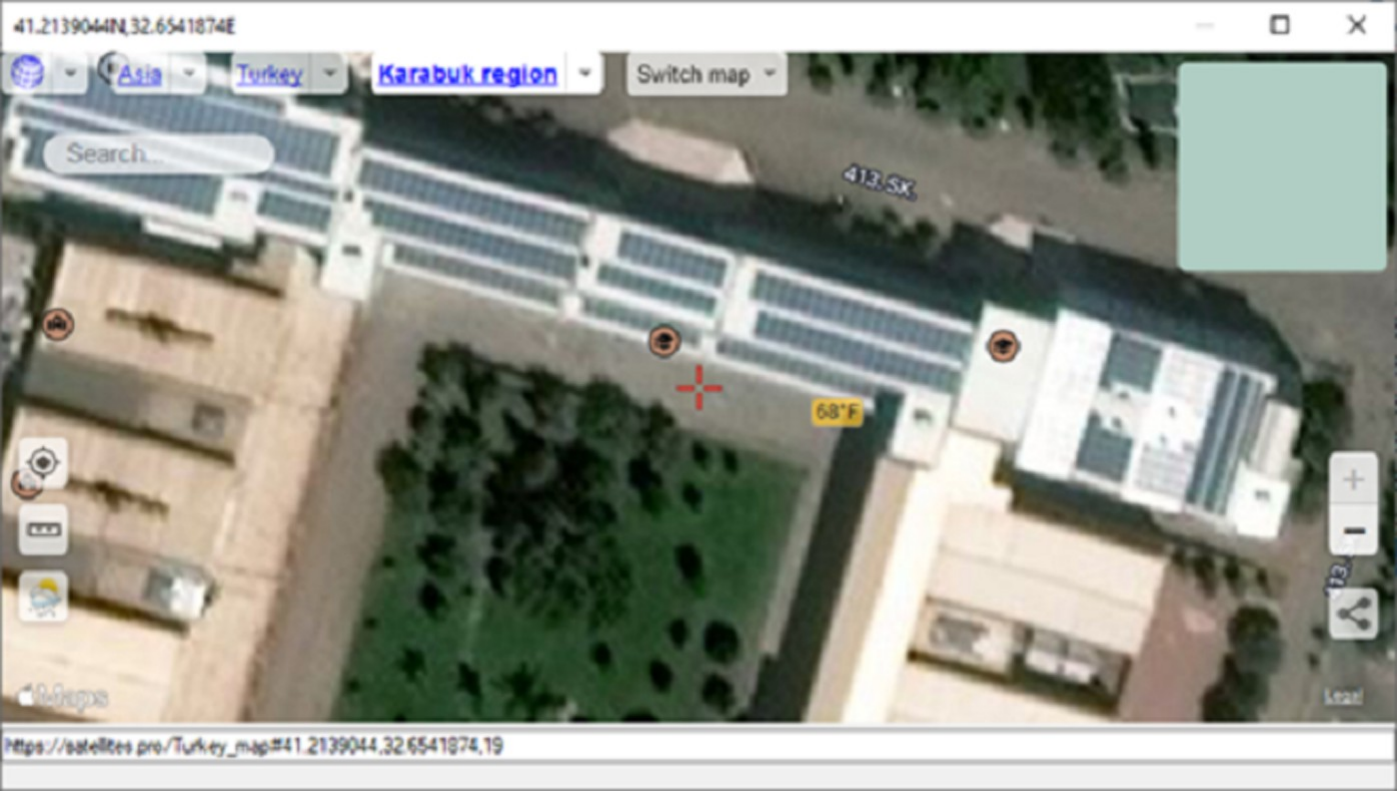
Real-time flight detection test.

Fig 11, illustrates a visual representation showcasing the regions identified as fire occurrences, serving as an illustrative example of the methodology used for presenting such image analyses.

**Fig 12:**
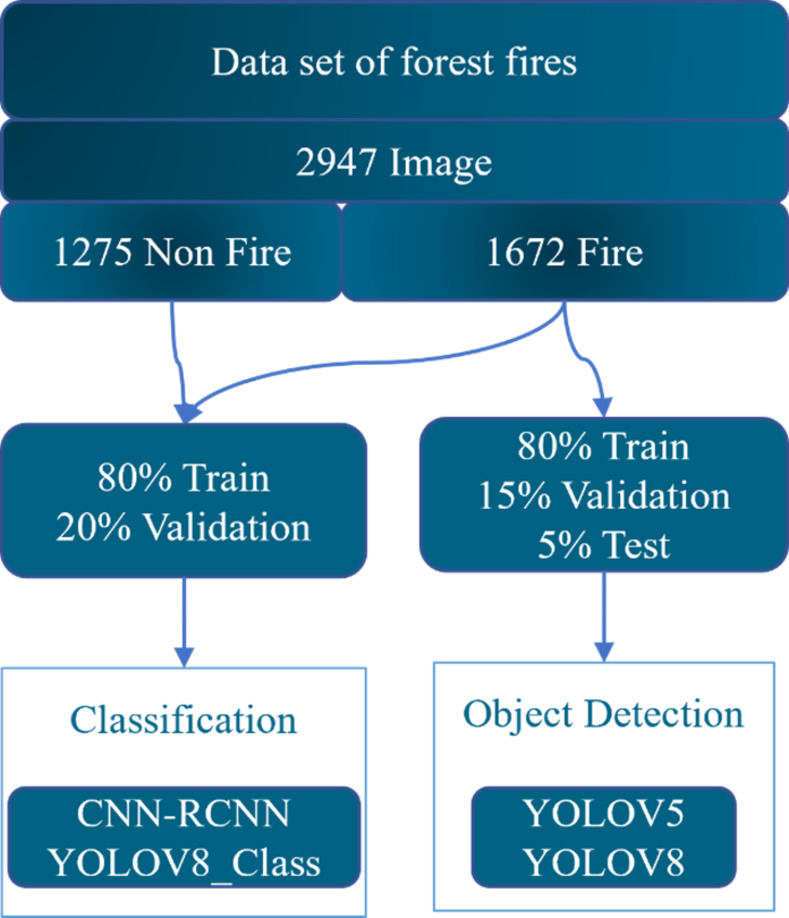
Image processing workflow.

**Fig 13:**
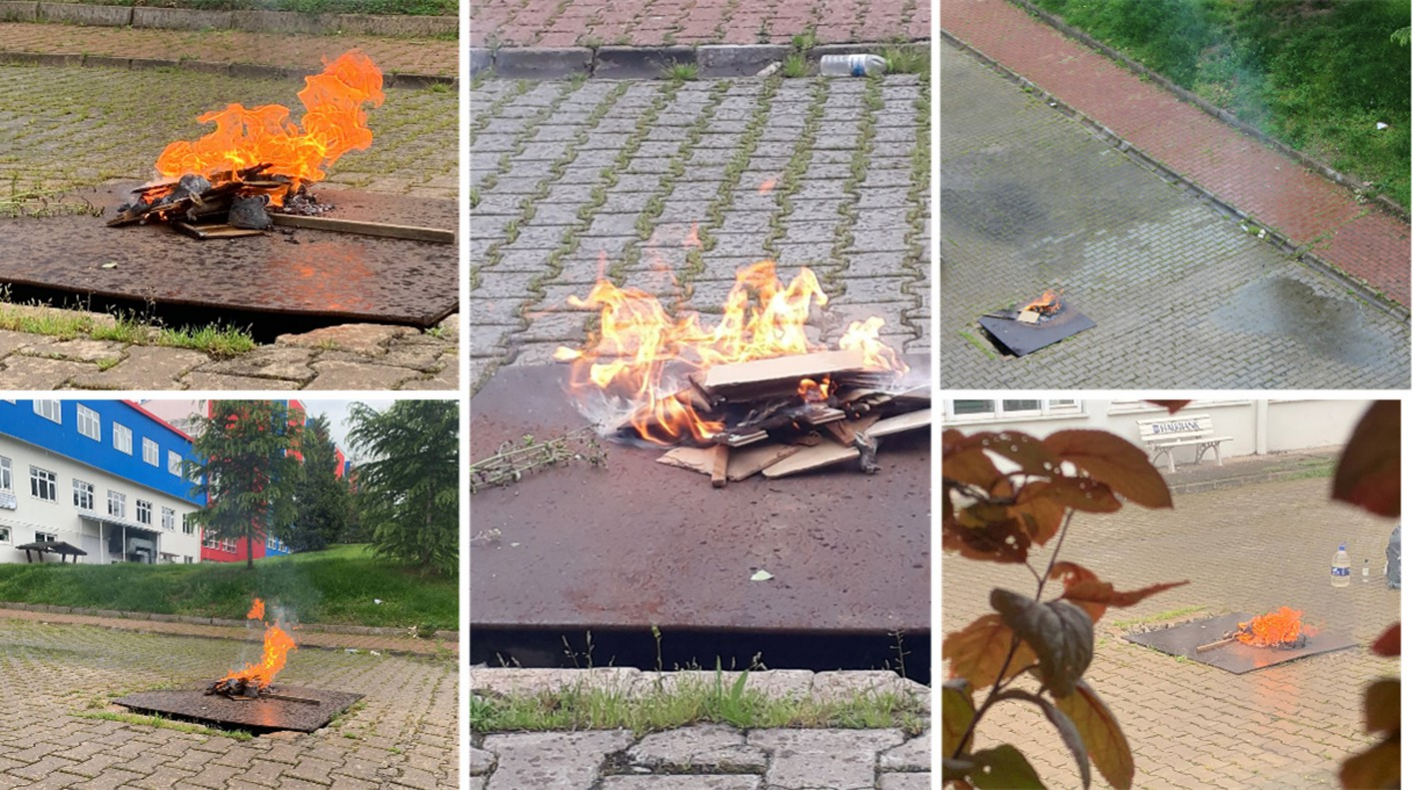
Forest fire dataset.

A diverse set of images depicting various types of forest fires, including large-scale forest fires, tree crown fires, and ground fires, was prepared to ensure the model’s ability to handle them. These images were collected, curated, and adjusted from multiple sources in alignment with the intended purpose of the study. The dataset utilized in this study comprised 2947 different images obtained from Mendeley [39] and personally collected data. The data was then divided into 80% for training, 5% for testing, and 15% for forest fire object detection validation. Fig 12, delineates the image processing workflow and its architectural structure as implemented for the models, ensuring image verification, and preventing duplication. For forest fire classifications, the dataset was divided into 80% for training and 20% for validation purposes. The models depicted in Fig 13 were based on this dataset [40].

## IV. Experimental results

**Fig 14:**
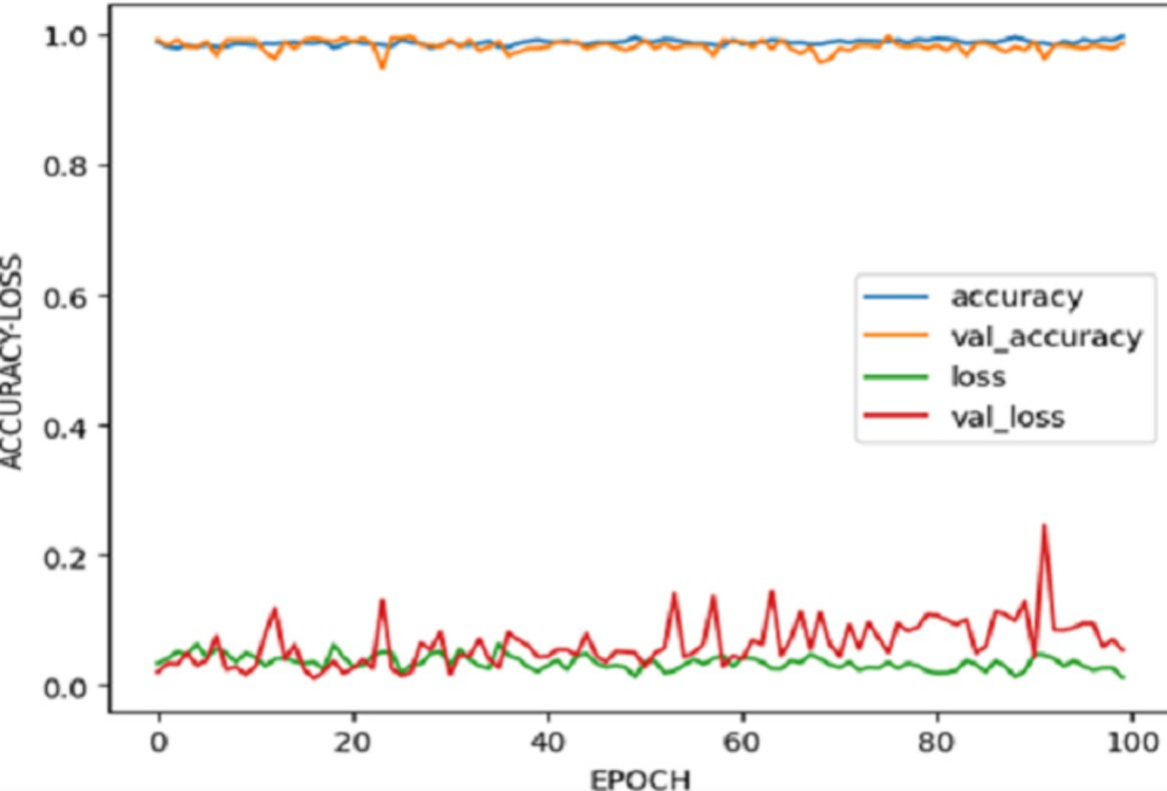
Evaluation matrix for CNN-RCNN.

The study’s outcomes unfolded through a multistage approach involving training and refinement. The training regimen for the CNN-RCNN model spanned across 500 epochs, with the model’s weights being consistently saved after each epoch. The conclusive epoch, reaching the 500th iteration (Epoch = 500), yielded the performance metrics detailed in Fig 14, encapsulating the evaluation matrix for the CNN-RCNN model.

**Fig 15:**
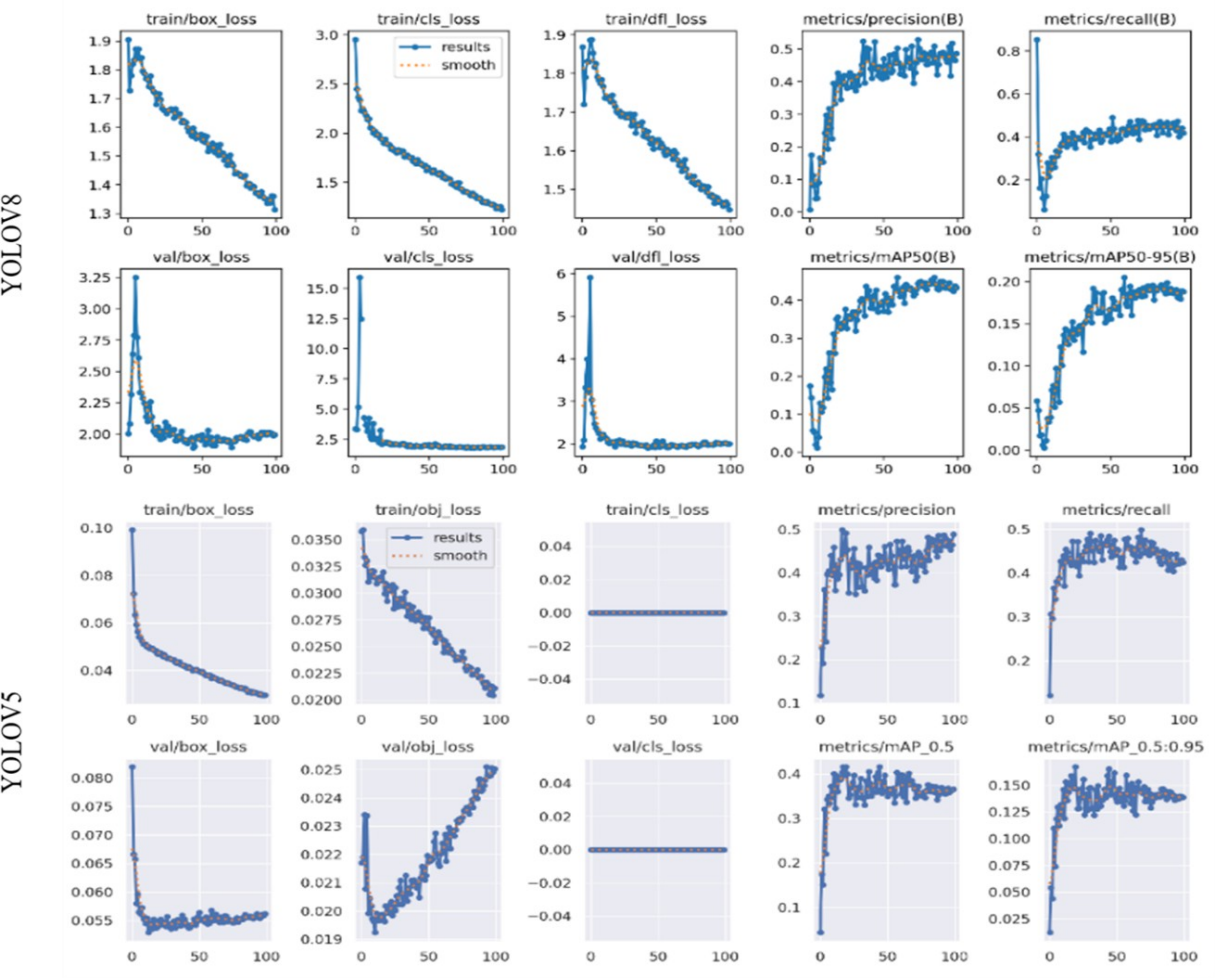
YOLOv8 vs YOLOv5 model training results.

Fig 15, showcases the outcomes derived from training the YOLOv8n and YOLOv5n models, offering visual representations in the form of curves that delineate the performance of both models. These graphical depictions allow for a comparative analysis of their behavior. While both models demonstrate similar performance levels, the plotted curves highlight YOLOv8’s superior performance, showcasing more favorable characteristics and yielding better results when contrasted with YOLOv5.

**Fig 16:**
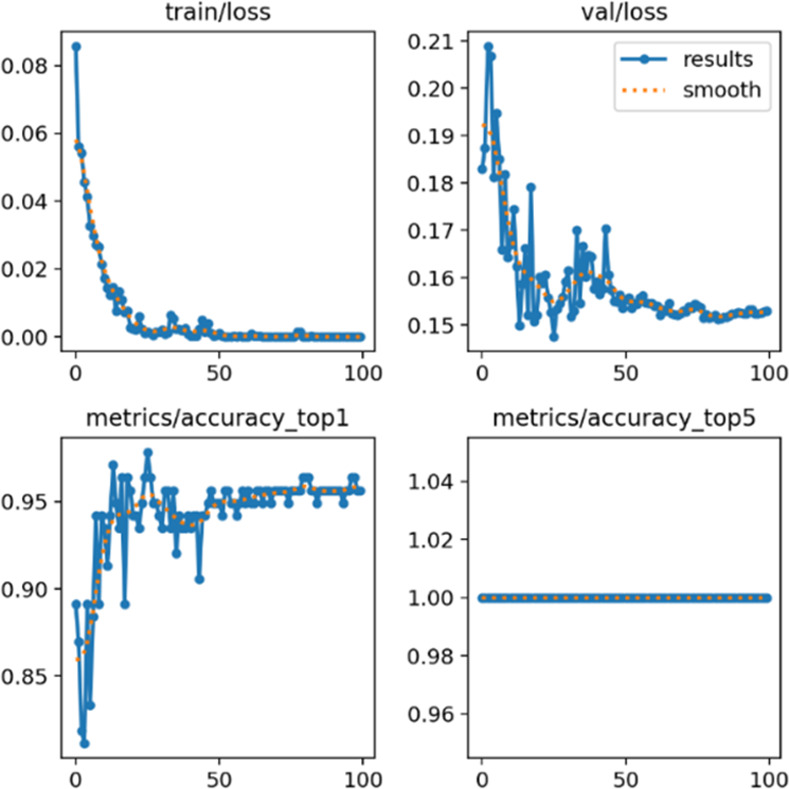
Training results of the YOLOv8 classification.

The YOLOv8 model, meticulously crafted for classification purposes, demonstrated exceptional performance characterized by superior accuracy and rapid predictive capabilities. In Fig 16, the representation exhibits a decline in error rates throughout the training and validation stages, concurrent with an upward trajectory in accuracy rates, emphasizing the model’s proficiency in learning and its capability to deliver precise classifications.

Table 4. presents the confusion matrix of the algorithms assessed within the study’s scope. Each algorithm underwent testing using an identical dataset that remained distinct from the dataset used in their training phases.

**Table 4:** The confusion matrix resulting from proposed models.

	Object Detection		Classification	
	YOLOv5	YOLOv8	YOLOv8	CNN-RCNN
TP	84	85	98	76
FP	6	7	6	5
TN	94	93	94	95
FN	16	15	2	2

The confusion matrix reports of each model reveal strikingly similar performance in classification and object detection, indicating closely comparable results among the models. Table 5. shows the confusion matrix report of each model.

**Table 5:** Confusion matrix reports for proposed models.

	Accuracy	Sensitivity	Specificity	Precision	F1_Skor	J_Skor	FPR
CNN-RCNN Classification	0.96	0.97	0.95	0.94	0.96	0.92	0.05
YOLOv8 Classification	0.96	0.98	0.94	0.94	0.96	0.92	0.06
YOLOv8n Object Detection	0.89	0.85	0.93	0.92	0.88	0.78	0.07
YOLOv5n Object Detection	0.89	0.84	0.94	0.93	0.88	0.78	0.06

In the domain of object detection, several terms are utilized to measure performance:

- mAP50: It is the average precision at 50% İntersection over Union (IoU) with bounding box truncation B (i.e., when an item is detected with 50% or higher accuracy, it is considered a positive) averaged across different classes.
- mAP50-95: The average precision at IoU thresholds ranging from 50% to 95% with bounding box truncation.
- Precision(B): The ratio of True Positive results (TP) to all positive results obtained with bounding box truncation.
- Recall: The number of true positive results detected within the bounding box is divided by the total number of ground truth positive targets.
- The PR (Precision-Recall) curve is used to evaluate the performance of detection and classification models in machine learning. This curve is plotted by calculating the precision and recall at different confidence thresholds to determine the detected objects. The values are then represented as a curve to illustrate the performance.

Table 6. illustrates the performance measurements of object detection models.

**Table 6:** Performance measurements of object detections for YOLOv5 VS YOLOv8.

Evaluation Metrics (Epoch = 100)					
	mAP50	mAP50-95	Precision	Recall	PR
Yolov8n	0.462	0.205	0.503	0.432	0.462
Yolov5n	0.365	0.138	0.448	0.424	0.416

**Fig 17:**
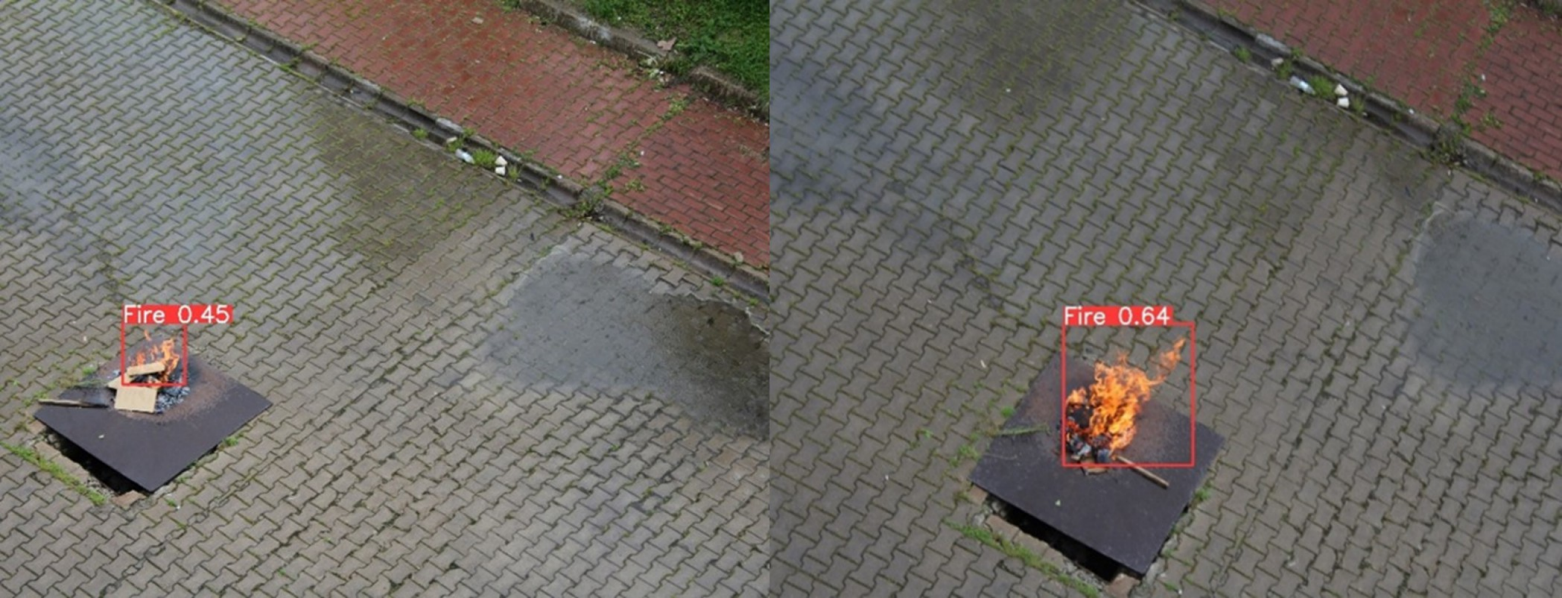
Real-time results for object detection.

The real-time implementation yielded satisfactory results; however, certain environmental conditions posed significant challenges to the proposed work. Additionally, the weight of the Jetson Nano emerged as a notable obstacle for our UAV, impacting both flight stability and motion. The intricate nature of object detection models, coupled with the challenges posed by dataset size and preprocessing steps, contributed to the lower accuracy observed in comparison to the classification models. The complexity of object detection tasks, as opposed to the more straightforward nature of classification models, further influenced this performance discrepancy. Notably, the real-time implementation revealed that classification models are computationally more efficient than their object detection counterparts. Consequently, our findings suggest that classification models are better suited for forest fire tasks within the context of our work. Fig 17, presents some real-time results for object detection models.

## V. Conclusions

This research has introduced an innovative early detection system for forest fires employing a UAV equipped with an NVIDIA Jetson Nano onboard and a camera. The study assessed the efficacy of YOLOv5 and YOLOv8 models for object detection, comparing them with the CNN-RCNN model for classification. Additionally, a ground station application was developed to collect data from the UAV. The findings revealed comparable accuracy between YOLOv5 and YOLOv8 for object detection, achieving an accuracy of approximately 89%. Furthermore, the comparison between YOLOv8 and CNN-RCNN for classification demonstrated an accuracy of about 96%. This suggests a promising avenue for utilizing deep learning models in UAV-based forest fire detection systems, showcasing their potential for high-accuracy early fire recognition. Further refinements and field implementations could lead to more robust and effective real-time fire monitoring systems.

## Supporting information

S1 Data (ZIP)
