# Supplementary figures and images for: Development of a deep learning-based surveillance system for forest fire detection and monitoring using UAV
Source: PLoS One. 2024 Mar 12;19(3):e0299058. doi: 10.1371/journal.pone.0299058 (PMC10931456; doi:10.1371/journal.pone.0299058)

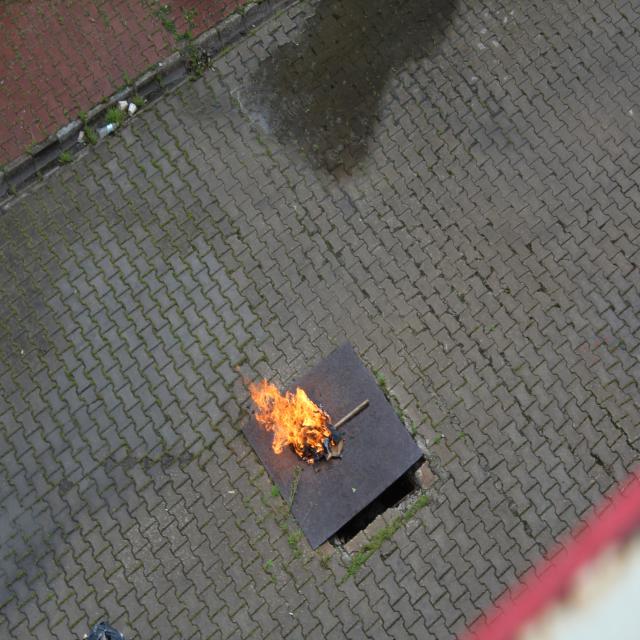

Supplement: S1 Data — (ZIP) [file pone.0299058.s001.zip › FireDataSetMinimum/13_JPG.rf.29b4f35f7d603f99ac1809b672896156 - Copy.jpg]

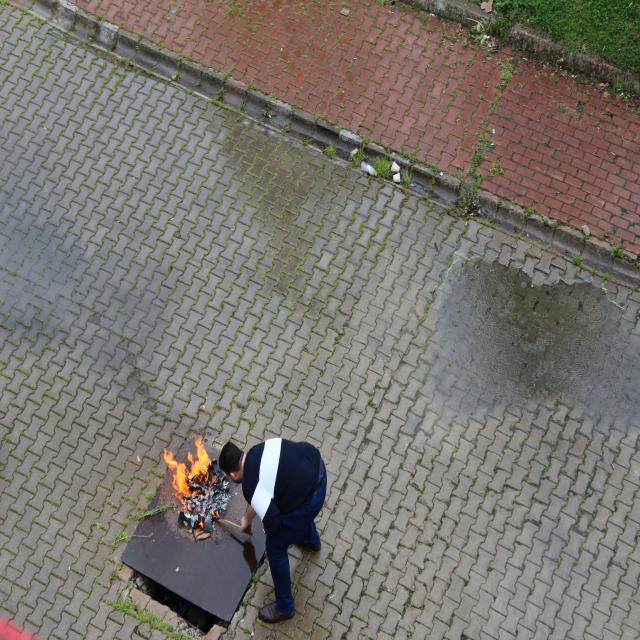

Supplement: S1 Data — (ZIP) [file pone.0299058.s001.zip › FireDataSetMinimum/155_JPG.rf.63bdce16a4e466f8d45f1f54ac31ff2f - Copy.jpg]

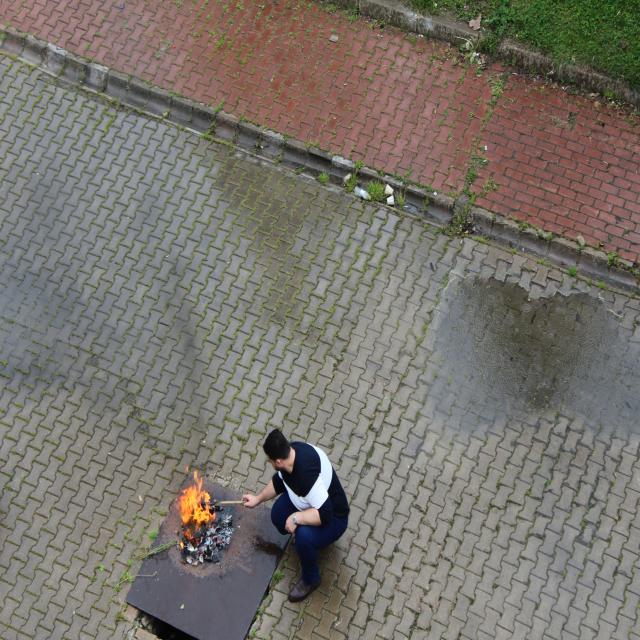

Supplement: S1 Data — (ZIP) [file pone.0299058.s001.zip › FireDataSetMinimum/171_JPG.rf.a55efd7581dd605e7965d21d9366c0a9 - Copy.jpg]

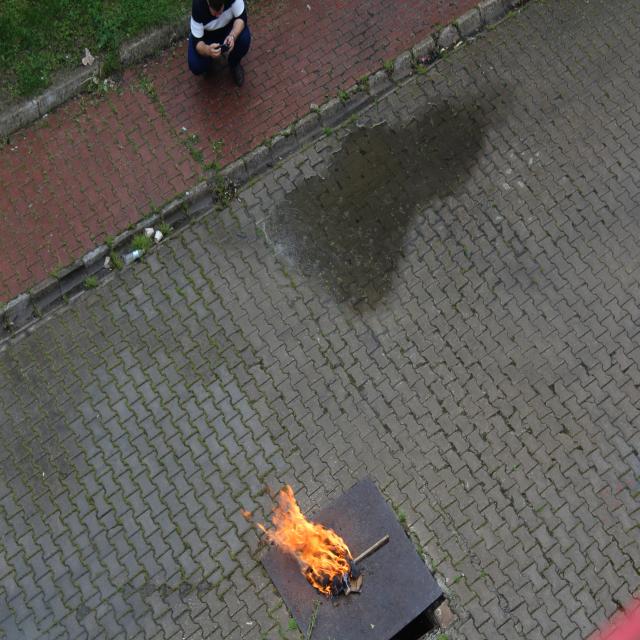

Supplement: S1 Data — (ZIP) [file pone.0299058.s001.zip › FireDataSetMinimum/17_JPG.rf.7b2aad13e5e4d0a269763f1968d2457f - Copy.jpg]

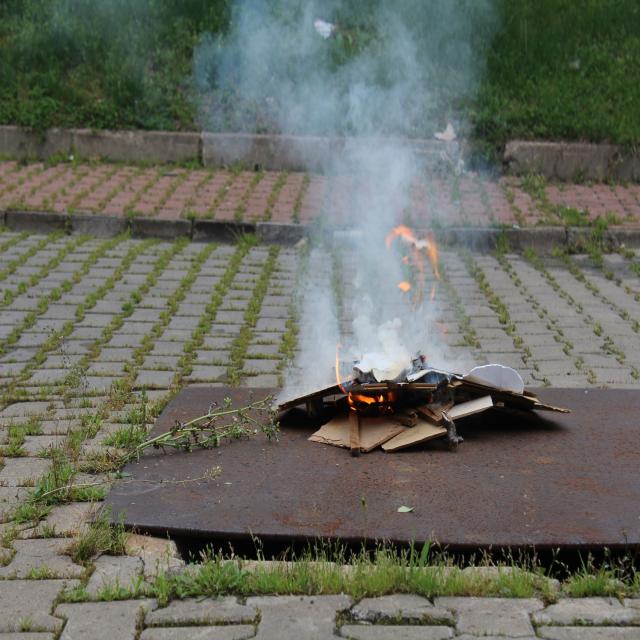

Supplement: S1 Data — (ZIP) [file pone.0299058.s001.zip › FireDataSetMinimum/1_JPG.rf.82be351019b363a690c1f9394144c4d9 - Copy.jpg]

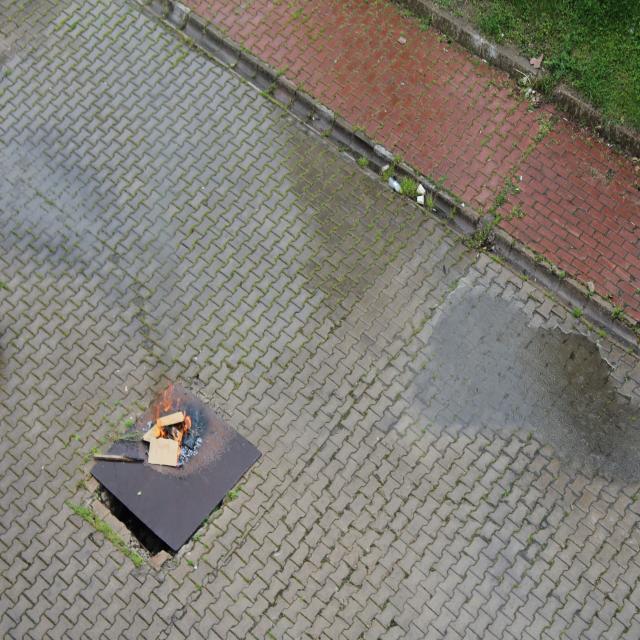

Supplement: S1 Data — (ZIP) [file pone.0299058.s001.zip › FireDataSetMinimum/225_JPG.rf.254cf4572703c816b917a463c4870d5c.jpg]

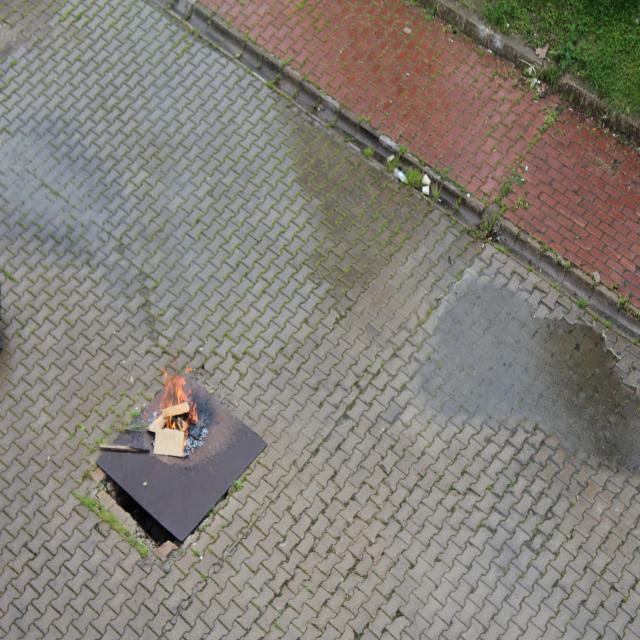

Supplement: S1 Data — (ZIP) [file pone.0299058.s001.zip › FireDataSetMinimum/233_JPG.rf.d21214682a26f926a0bcece9483badb3 - Copy.jpg]

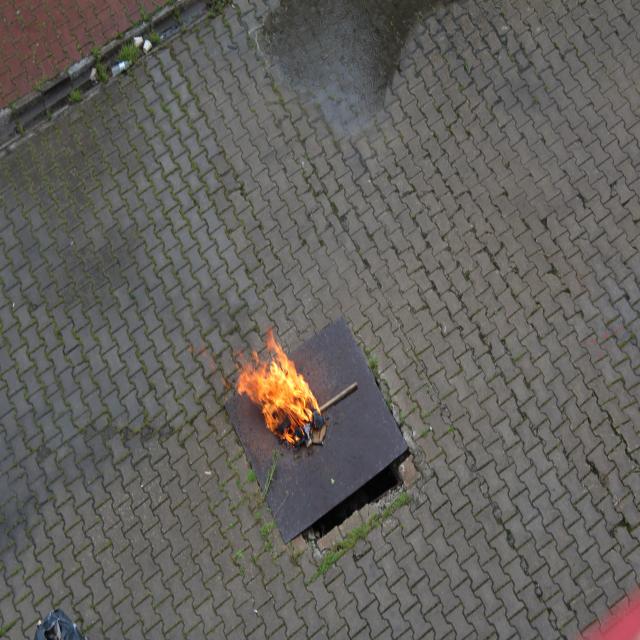

Supplement: S1 Data — (ZIP) [file pone.0299058.s001.zip › FireDataSetMinimum/23_JPG.rf.78cdab13cee2666bafa6a4f9917bb3cc.jpg]

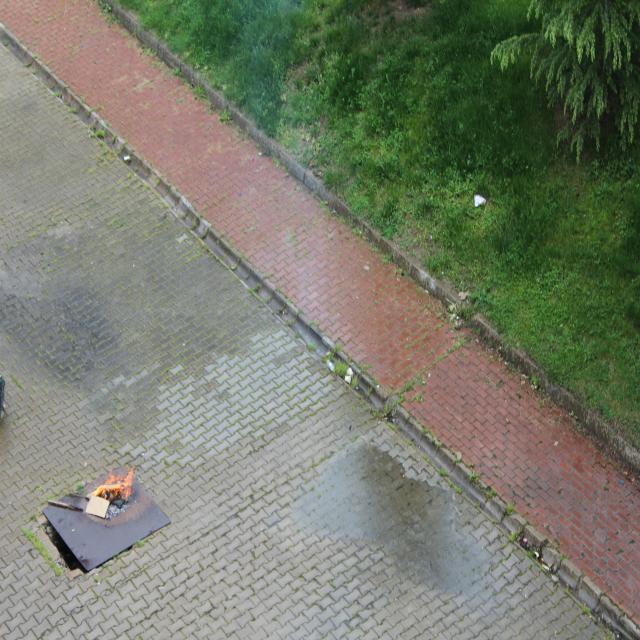

Supplement: S1 Data — (ZIP) [file pone.0299058.s001.zip › FireDataSetMinimum/249_JPG.rf.fedc7e140b6fc9021ff8c3e309535103.jpg]

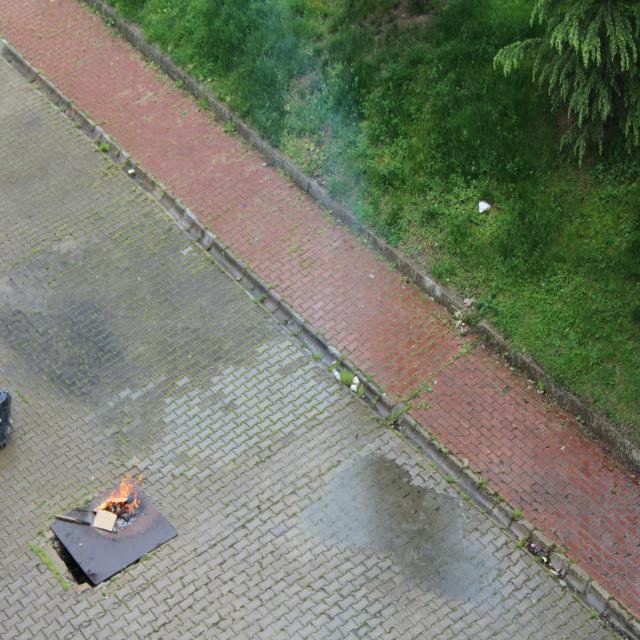

Supplement: S1 Data — (ZIP) [file pone.0299058.s001.zip › FireDataSetMinimum/253_JPG.rf.c08d94111920ff4f0ff80ab2e40c819e - Copy.jpg]

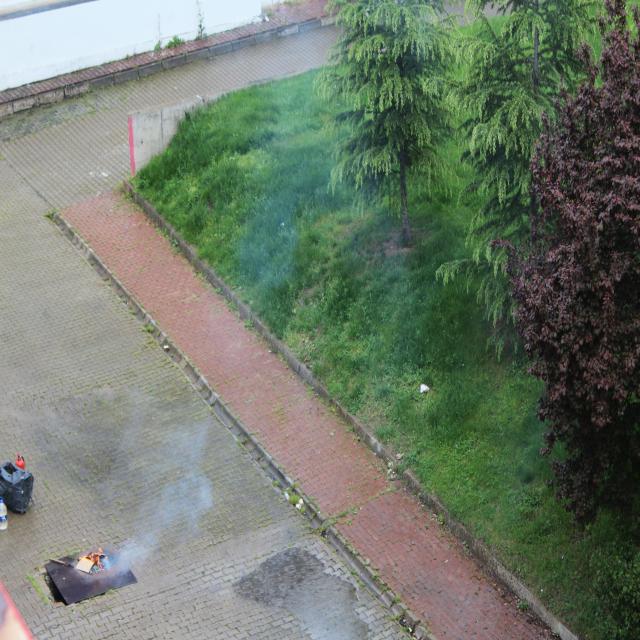

Supplement: S1 Data — (ZIP) [file pone.0299058.s001.zip › FireDataSetMinimum/257_JPG.rf.81d32e395f8de0cfb5bcf8ea2143d6f7.jpg]

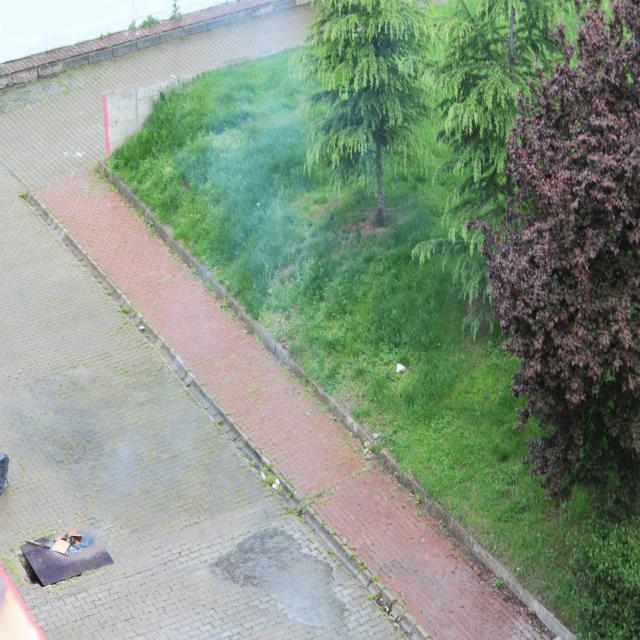

Supplement: S1 Data — (ZIP) [file pone.0299058.s001.zip › FireDataSetMinimum/263_JPG.rf.ed8abb33a2a145cddf5fab681c1feed4 - Copy.jpg]

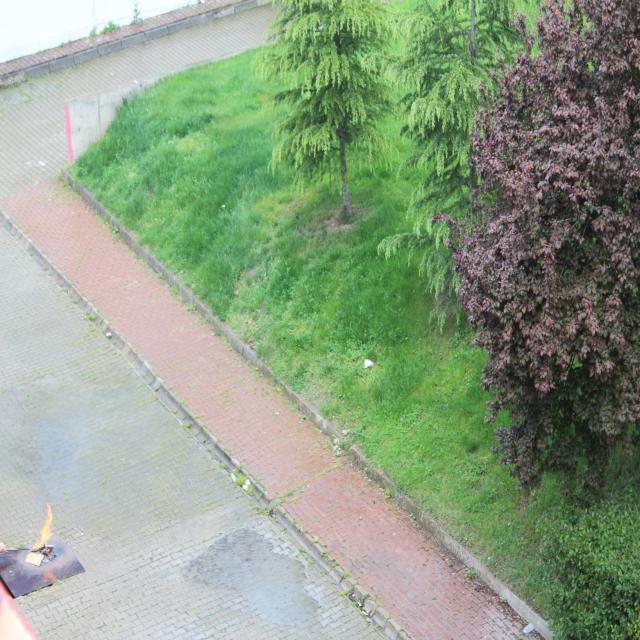

Supplement: S1 Data — (ZIP) [file pone.0299058.s001.zip › FireDataSetMinimum/265_JPG.rf.15958e35fbf97314e8d5823b32df784c - Copy.jpg]

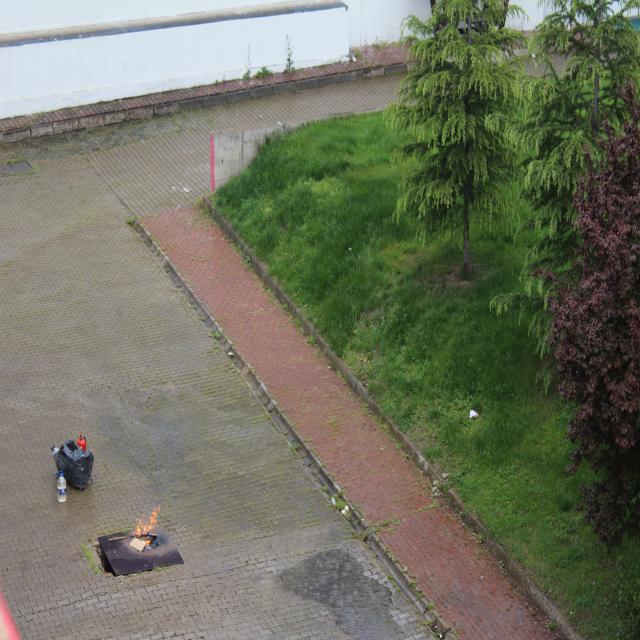

Supplement: S1 Data — (ZIP) [file pone.0299058.s001.zip › FireDataSetMinimum/281_JPG.rf.2736e80e4b714ffbf4439b6d5a750cff.jpg]

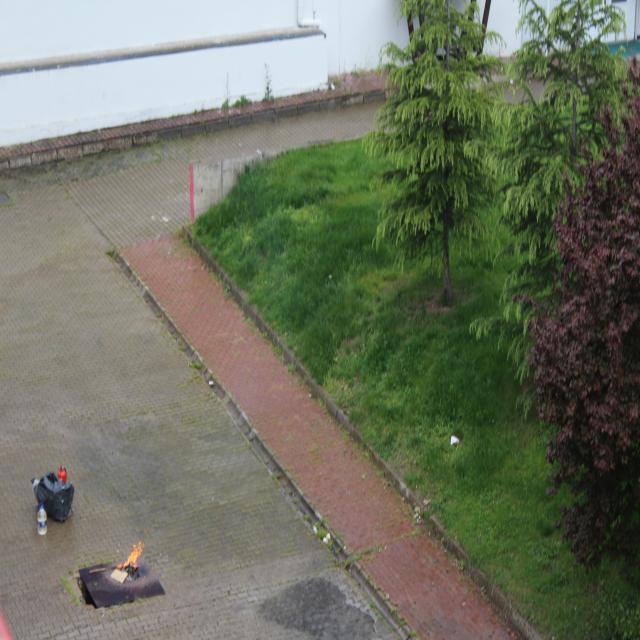

Supplement: S1 Data — (ZIP) [file pone.0299058.s001.zip › FireDataSetMinimum/303_JPG.rf.a2d548c721fbe497f777dad32eae242b.jpg]

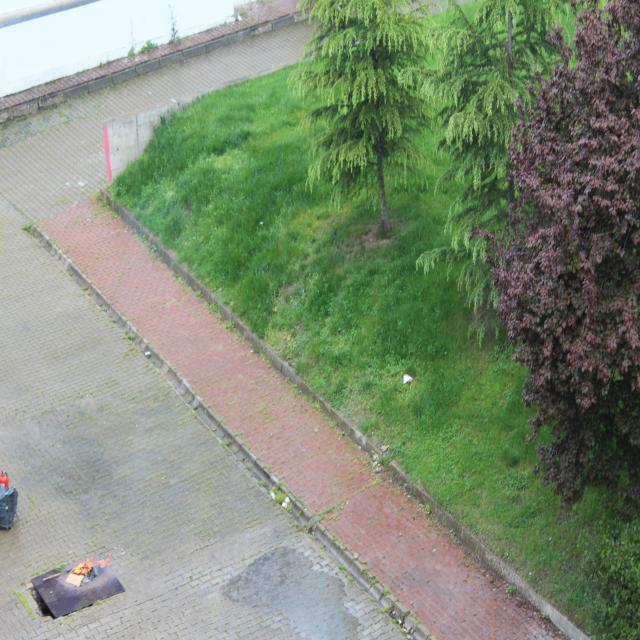

Supplement: S1 Data — (ZIP) [file pone.0299058.s001.zip › FireDataSetMinimum/323_JPG.rf.aa5120d6756d57128a0df5b11ef8643a.jpg]

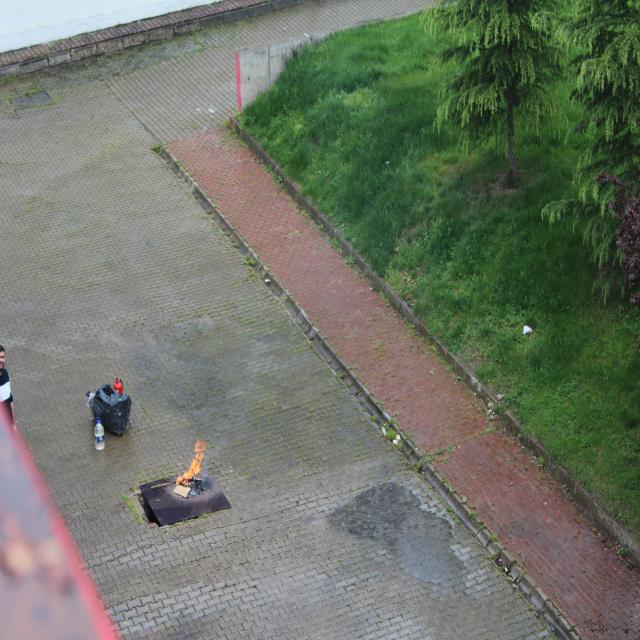

Supplement: S1 Data — (ZIP) [file pone.0299058.s001.zip › FireDataSetMinimum/333_JPG.rf.5b1600f8f74f60b5c6e36f1af6dd2b90.jpg]

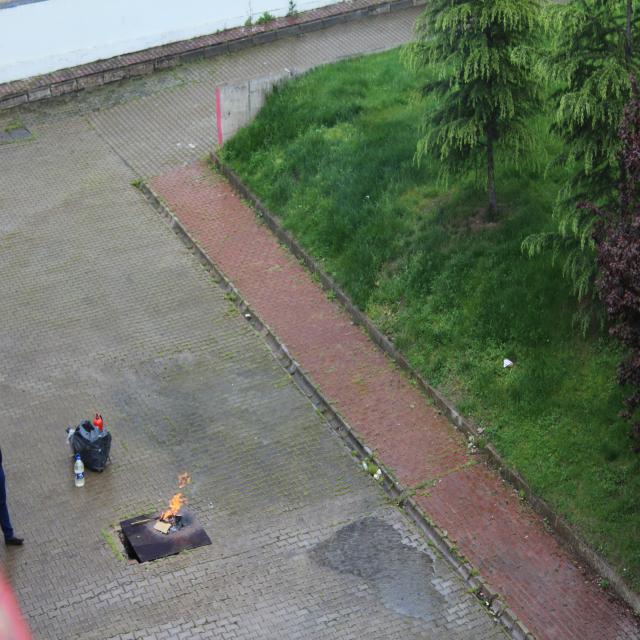

Supplement: S1 Data — (ZIP) [file pone.0299058.s001.zip › FireDataSetMinimum/343_JPG.rf.e81fe94938f11b10fdadcef1f80a7a6b.jpg]

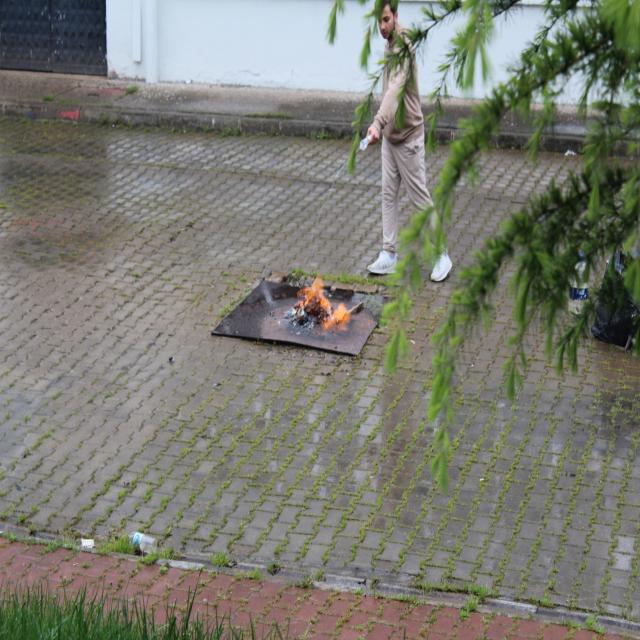

Supplement: S1 Data — (ZIP) [file pone.0299058.s001.zip › FireDataSetMinimum/351_JPG.rf.c24cbd1bd5906d262cb688aed2f03248.jpg]

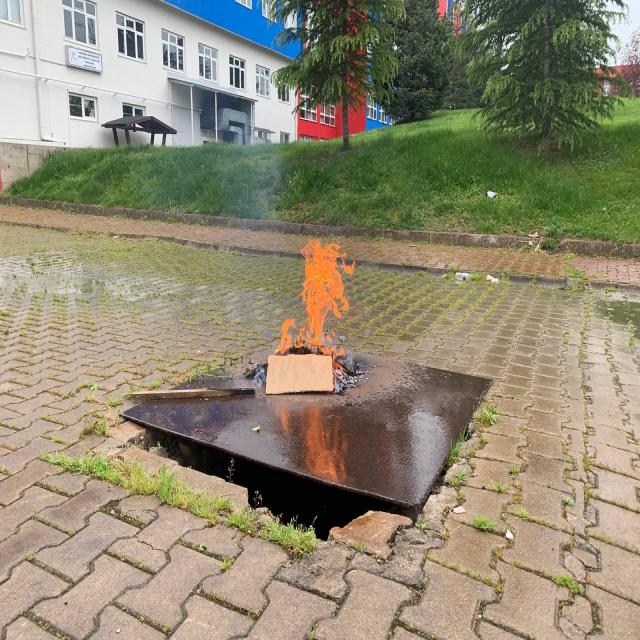

Supplement: S1 Data — (ZIP) [file pone.0299058.s001.zip › FireDataSetMinimum/371_jpg.rf.657979f8d5c284e14996ac1d5a3c8710.jpg]

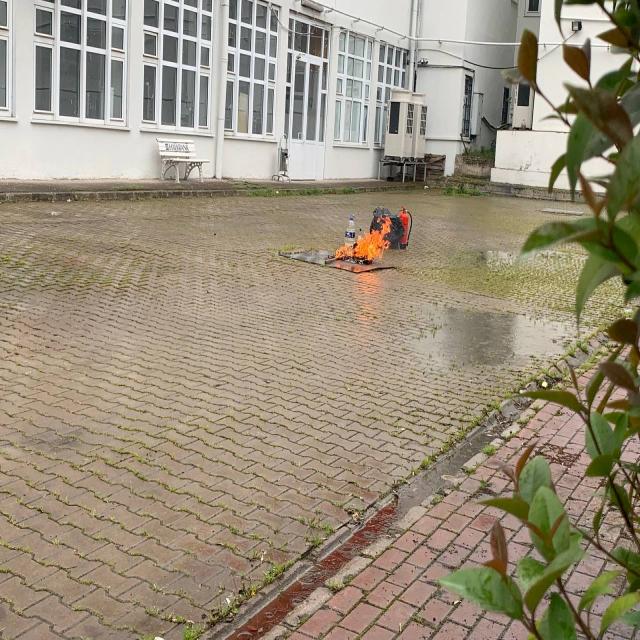

Supplement: S1 Data — (ZIP) [file pone.0299058.s001.zip › FireDataSetMinimum/375_jpg.rf.06b5acd42376d12d30cbdd5c4560d30c.jpg]

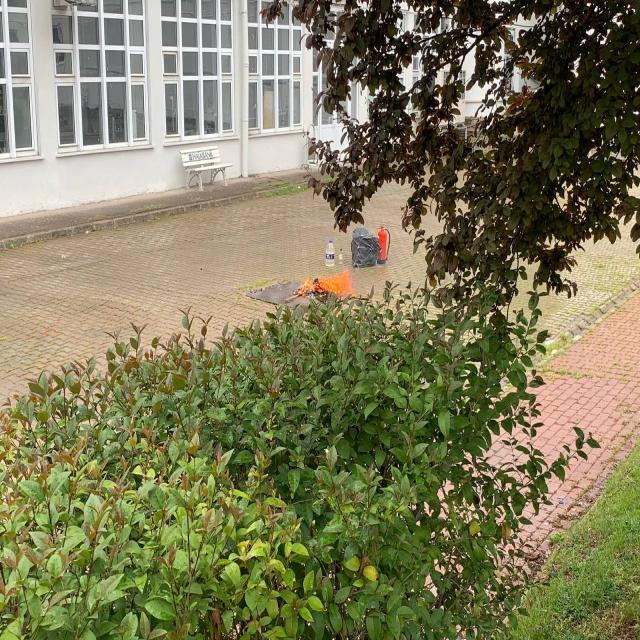

Supplement: S1 Data — (ZIP) [file pone.0299058.s001.zip › FireDataSetMinimum/377_jpg.rf.8867d35376d7460068933540fdb08ddd.jpg]

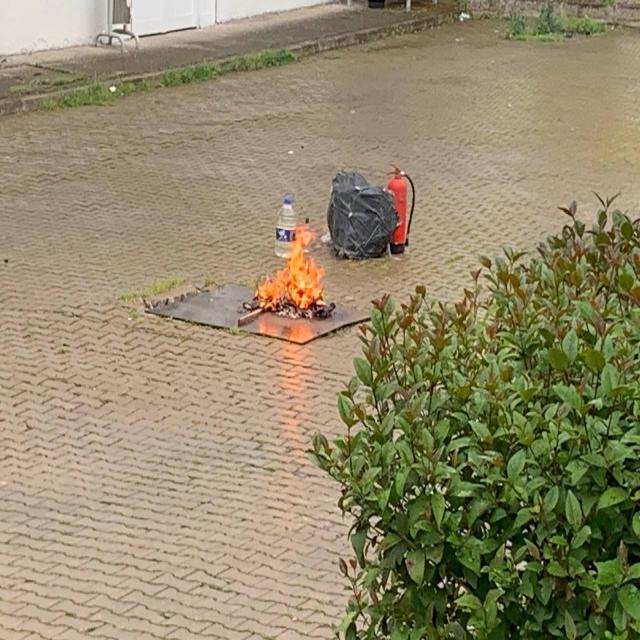

Supplement: S1 Data — (ZIP) [file pone.0299058.s001.zip › FireDataSetMinimum/379_jpg.rf.eb071917d7cc2e5650ecfa62f44f71d5.jpg]

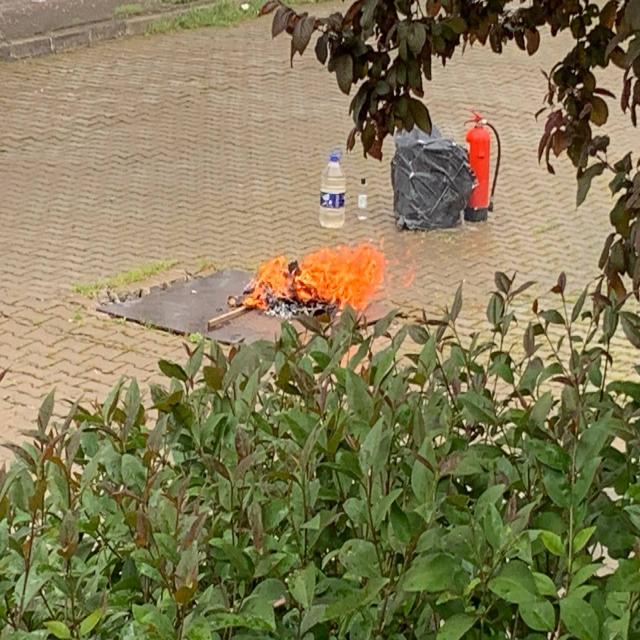

Supplement: S1 Data — (ZIP) [file pone.0299058.s001.zip › FireDataSetMinimum/383_jpg.rf.0cf58d995267d834e13d5a67a6803583.jpg]

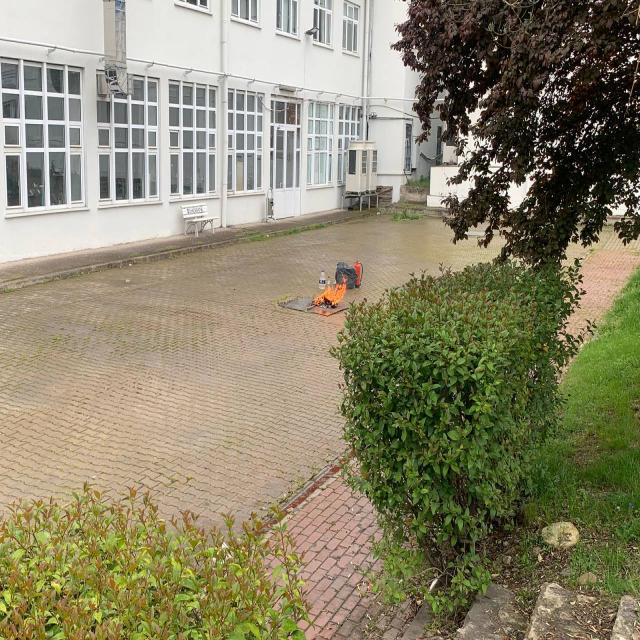

Supplement: S1 Data — (ZIP) [file pone.0299058.s001.zip › FireDataSetMinimum/385_jpg.rf.3f267793f970ef35669aff9eec7ccb3b.jpg]

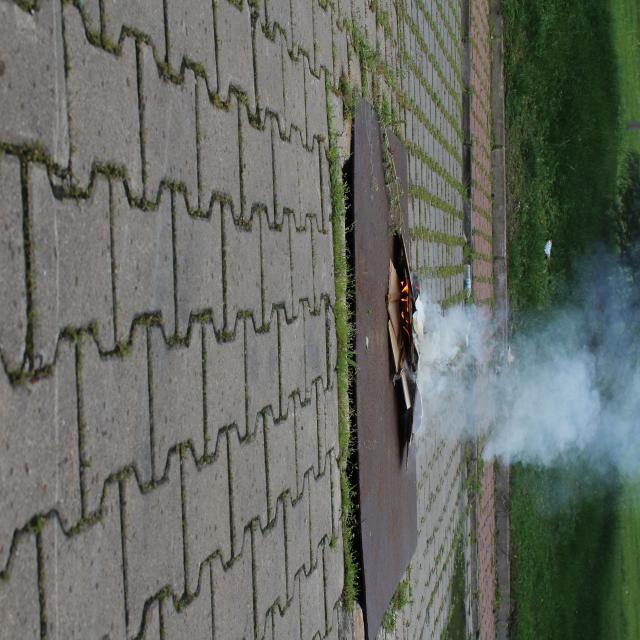

Supplement: S1 Data — (ZIP) [file pone.0299058.s001.zip › FireDataSetMinimum/3_JPG.rf.48889bb592371a826b2ceb2c06d48a69 - Copy.jpg]

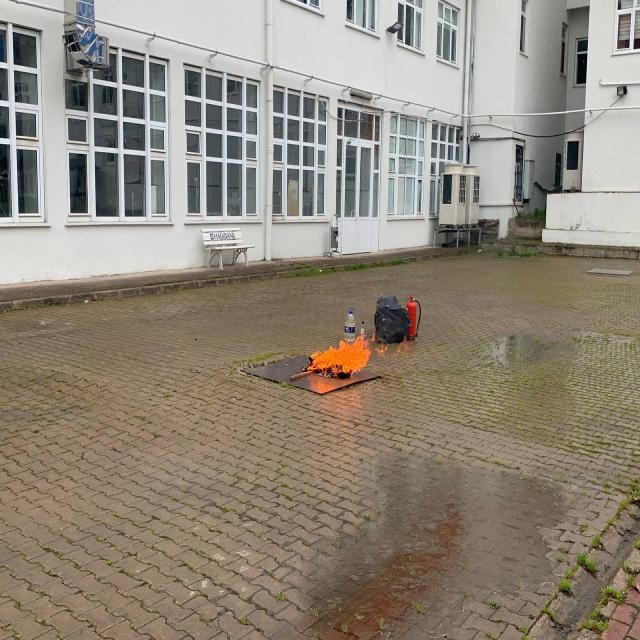

Supplement: S1 Data — (ZIP) [file pone.0299058.s001.zip › FireDataSetMinimum/407_jpg.rf.d9240a1b14a4bb5d2ba6720d11065035.jpg]

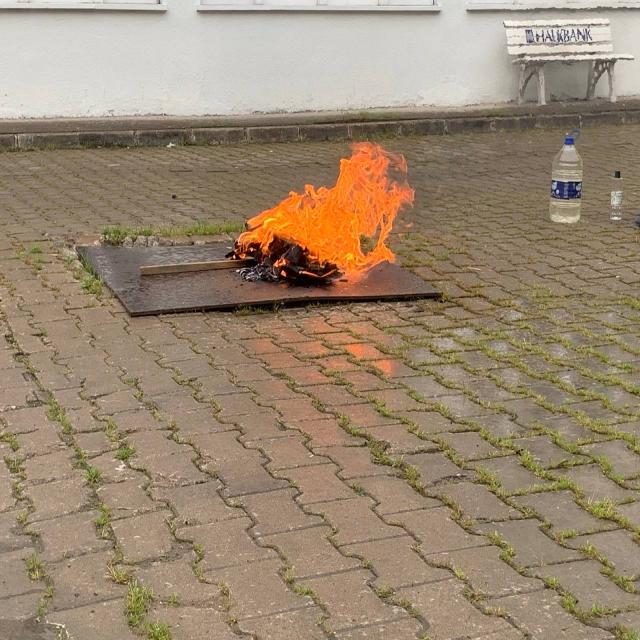

Supplement: S1 Data — (ZIP) [file pone.0299058.s001.zip › FireDataSetMinimum/409_jpg.rf.5c27011b5240d975ea6690eb3bbc3abf.jpg]

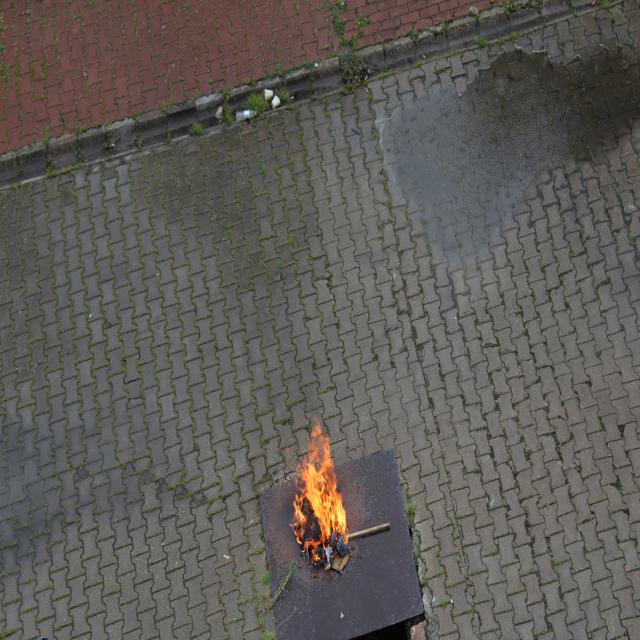

Supplement: S1 Data — (ZIP) [file pone.0299058.s001.zip › FireDataSetMinimum/41_JPG.rf.517f8afd6337c9d4ca80a304ebf2fd11 - Copy.jpg]

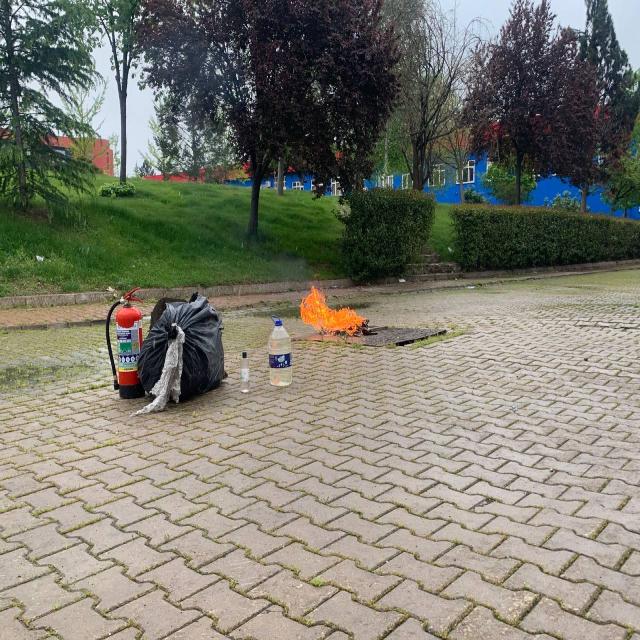

Supplement: S1 Data — (ZIP) [file pone.0299058.s001.zip › FireDataSetMinimum/421_jpg.rf.48ea0bf3a0001a9ef85d83febec6a749.jpg]

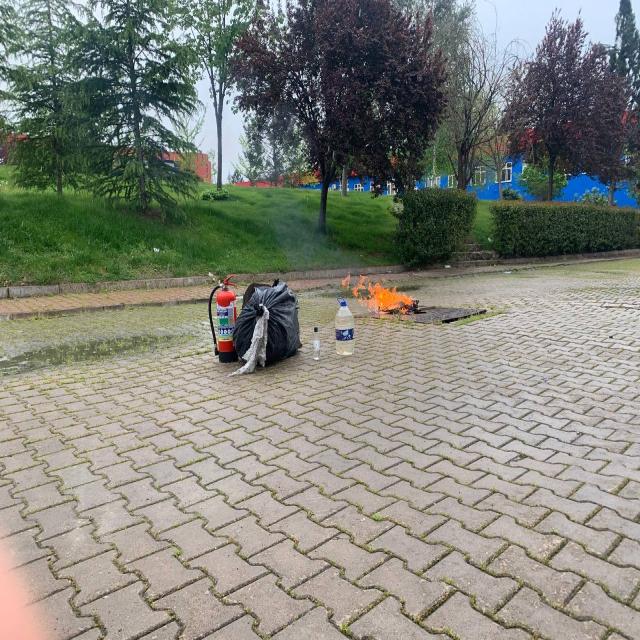

Supplement: S1 Data — (ZIP) [file pone.0299058.s001.zip › FireDataSetMinimum/429_jpg.rf.ad6013e1a2547063f615f01f584989f5.jpg]

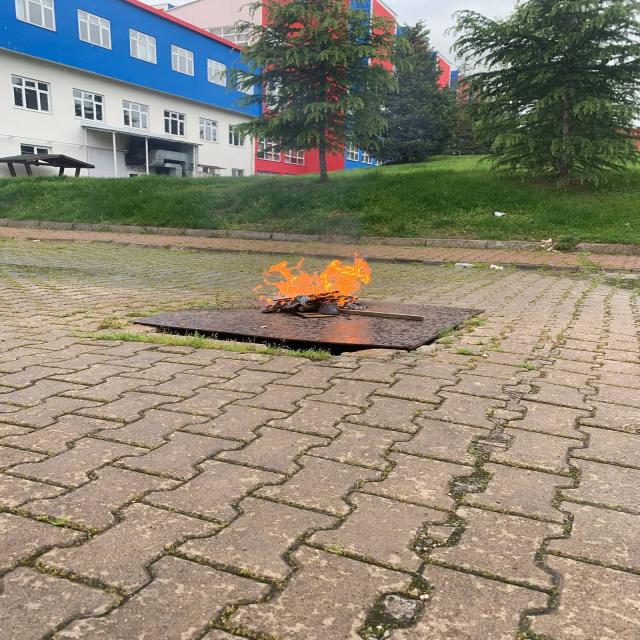

Supplement: S1 Data — (ZIP) [file pone.0299058.s001.zip › FireDataSetMinimum/431_jpg.rf.469bfd9a40d7a86a78909a7742b23a90.jpg]

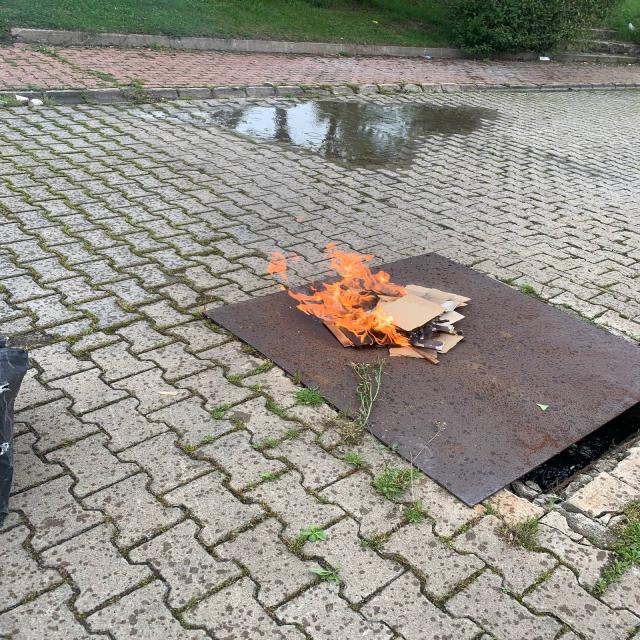

Supplement: S1 Data — (ZIP) [file pone.0299058.s001.zip › FireDataSetMinimum/449_jpg.rf.f5f243ae0289c1e29424451ae0fe0d80.jpg]

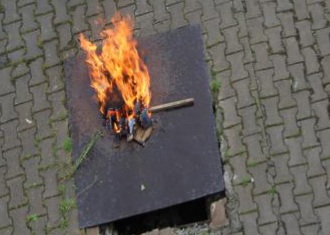

Supplement: S1 Data — (ZIP) [file pone.0299058.s001.zip › FireDataSetMinimum/45_JPG.rf.96386cc09498ff46352d61280a771945 - Copy.jpg]

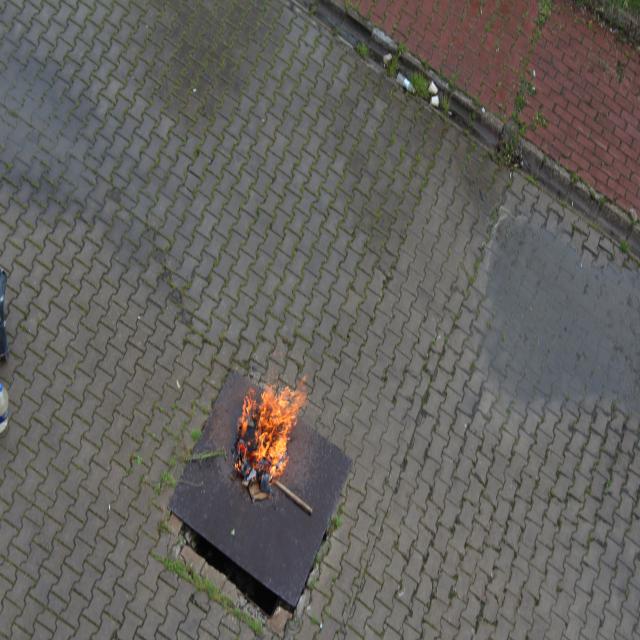

Supplement: S1 Data — (ZIP) [file pone.0299058.s001.zip › FireDataSetMinimum/47_JPG.rf.f20d0842200957d3095fd97e7cb0a4a3.jpg]

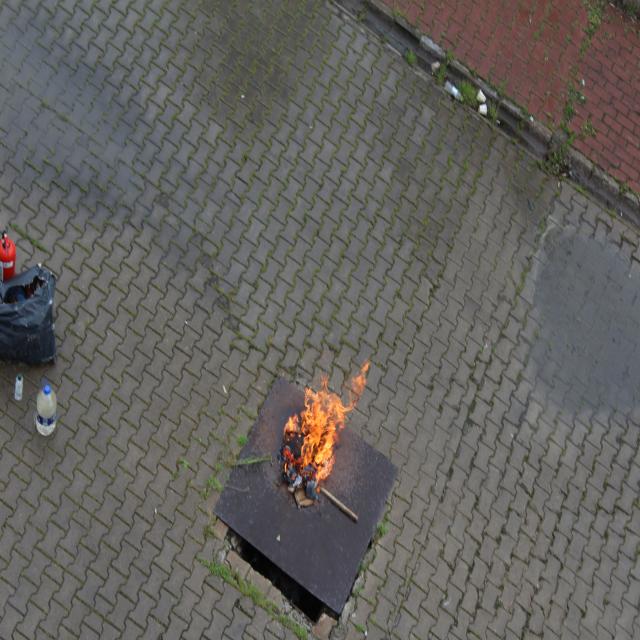

Supplement: S1 Data — (ZIP) [file pone.0299058.s001.zip › FireDataSetMinimum/49_JPG.rf.f9691debfc619bd08d879ef8aad26c7f - Copy.jpg]

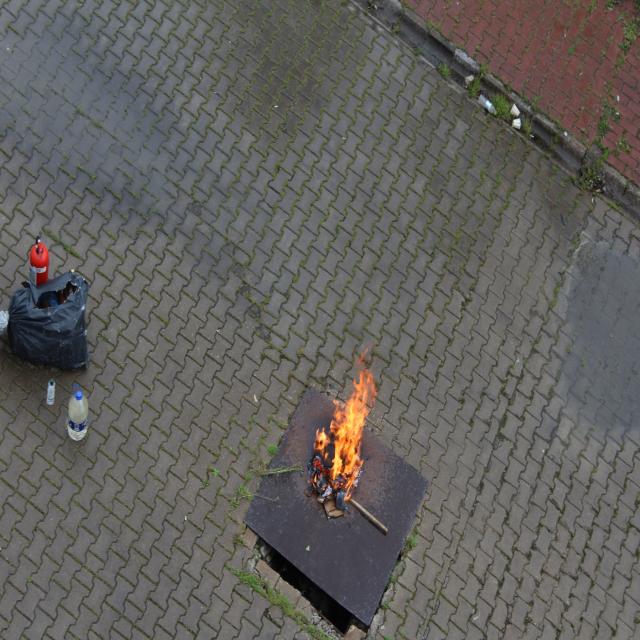

Supplement: S1 Data — (ZIP) [file pone.0299058.s001.zip › FireDataSetMinimum/53_JPG.rf.9c43508464994fe1440488f427346e28 - Copy.jpg]

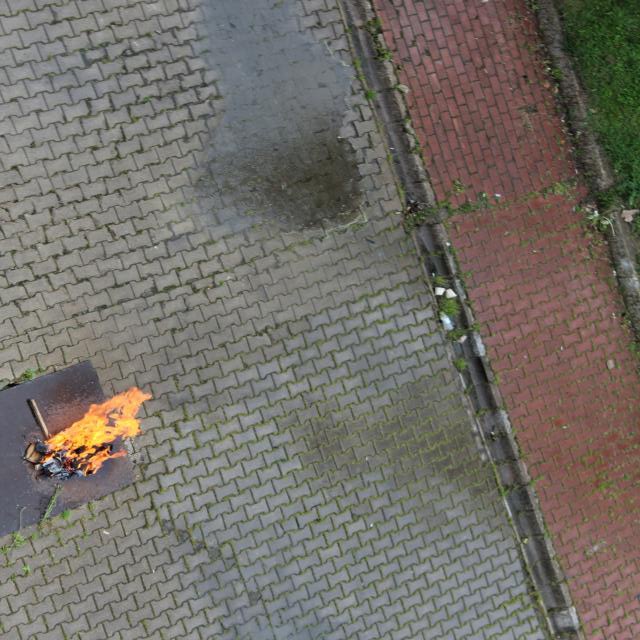

Supplement: S1 Data — (ZIP) [file pone.0299058.s001.zip › FireDataSetMinimum/61_JPG.rf.834529f4e7cbfe1ac1455c7fb2aa1edb - Copy.jpg]

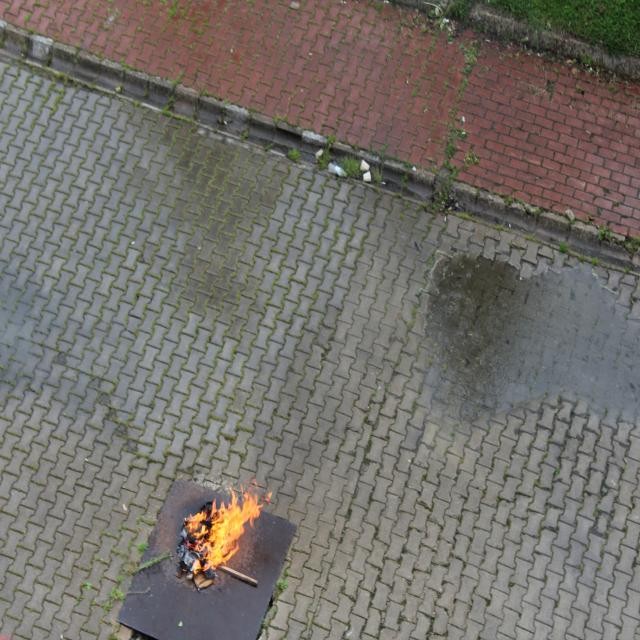

Supplement: S1 Data — (ZIP) [file pone.0299058.s001.zip › FireDataSetMinimum/67_JPG.rf.c27004c60721f98765734132a2a91035 - Copy.jpg]

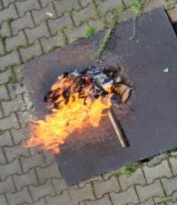

Supplement: S1 Data — (ZIP) [file pone.0299058.s001.zip › FireDataSetMinimum/69_JPG.rf.f8a94c1e3617305a0432bfaf1aeeb65d - Copy.jpg]

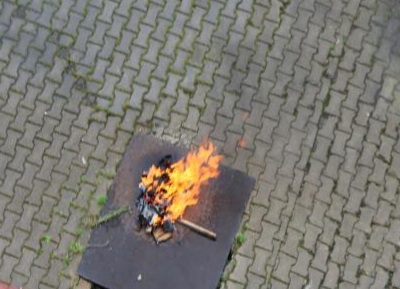

Supplement: S1 Data — (ZIP) [file pone.0299058.s001.zip › FireDataSetMinimum/75_JPG.rf.a09a35223a4370bb43f3932d1061c481.jpg]

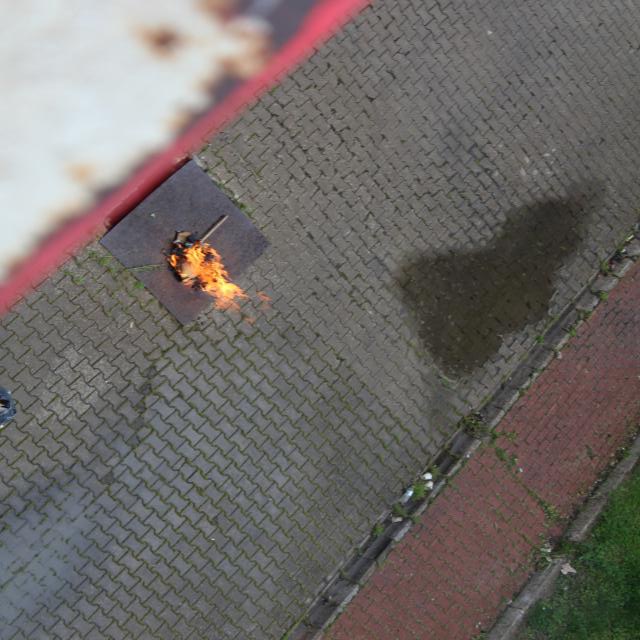

Supplement: S1 Data — (ZIP) [file pone.0299058.s001.zip › FireDataSetMinimum/7_JPG.rf.abb0242db00ec4f6376ae22429e22523 - Copy.jpg]

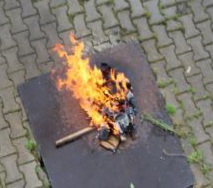

Supplement: S1 Data — (ZIP) [file pone.0299058.s001.zip › FireDataSetMinimum/81_JPG.rf.befd26df4e2f4dbb2faae0362609fad1 - Copy.jpg]

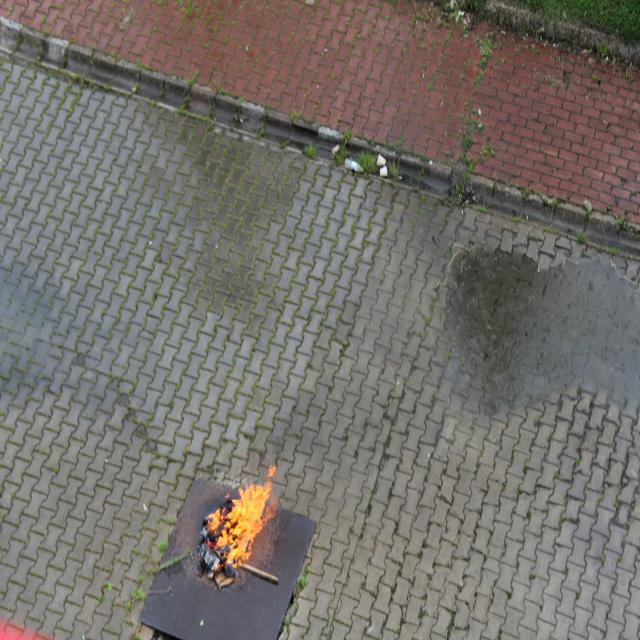

Supplement: S1 Data — (ZIP) [file pone.0299058.s001.zip › FireDataSetMinimum/85_JPG.rf.d8ecf1e1ef9191385bff95dcbca3a39b - Copy.jpg]

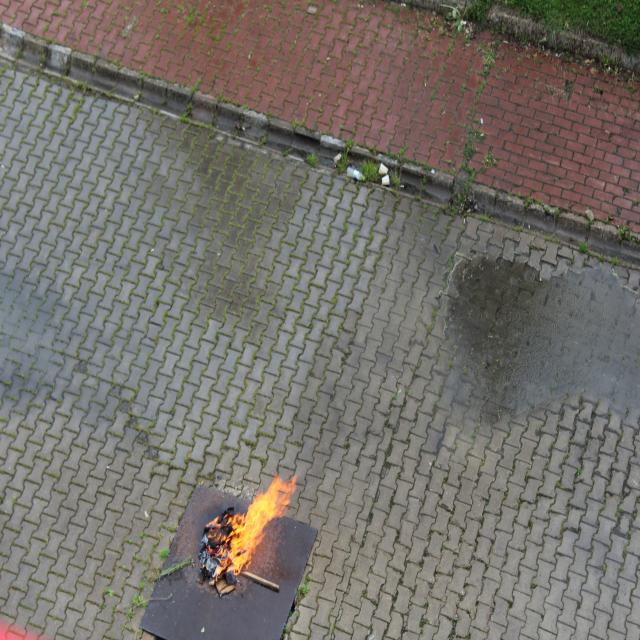

Supplement: S1 Data — (ZIP) [file pone.0299058.s001.zip › FireDataSetMinimum/87_JPG.rf.6314c593e835756ce2fb403454f3bfba - Copy.jpg]
